# Evaluating Serpentinization as a Source of Phosphite to Microbial Communities in Hydrothermal Vents

**DOI:** 10.1111/gbi.70016

**Published:** 2025-03-25

**Authors:** Joanne S. Boden, Sanjoy M. Som, William J. Brazelton, Rika E. Anderson, Eva E. Stüeken

**Affiliations:** ^1^ School of Earth and Environmental Sciences University of St. Andrews St. Andrews UK; ^2^ Blue Marble Space Institute of Science Seattle Washington USA; ^3^ Space Science and Astrobiology Division, Exobiology Branch NASA Ames Research Center Mountain View California USA; ^4^ School of Biological Sciences University of Utah Salt Lake City Utah USA; ^5^ Department of Biology Carleton College Northfield Minnesota USA

**Keywords:** Archean, geothermal, hydrothermal vent, metagenomics, phosphite, phosphorus, serpentinization

## Abstract

Previous studies have documented the presence of phosphite, a reduced and highly soluble form of phosphorus, in serpentinites, which has led to the hypothesis that serpentinizing hydrothermal vents could have been an important source of bioavailable phosphorus for early microbial communities in the Archean. Here, we test this hypothesis by evaluating the genomic hallmarks of phosphorus usage in microbial communities living in modern hydrothermal vents with and without influence from serpentinization. These genomic analyses are combined with results from a geochemical model that calculates phosphorus speciation during serpentinization as a function of temperature, water:rock ratio, and lithology at thermodynamic equilibrium. We find little to no genomic evidence of phosphite use in serpentinizing environments at the Voltri Massif or the Von Damm hydrothermal field at the Mid Cayman Rise, but relatively more in the Lost City hydrothermal field, Coast Range Ophiolite Microbial Observatory, The Cedars, and chimney samples from Old City hydrothermal field and Prony Bay hydrothermal field, as well as in the non‐serpentinizing hydrothermal vents at Axial Seamount. Geochemical modeling shows that phosphite production is favored at ca 275°C–325°C and low water:rock ratios, which may explain previous observations of phosphite in serpentinite rocks; however, most of the initial phosphate is trapped in apatite during serpentinization, suppressing the absolute phosphite yield. As a result, phosphite from serpentinizing vents could have supported microbial growth around olivine minerals in chimney walls and suspended aggregates, but it is unlikely to have fueled substantial primary productivity in diffusely venting fluids during life's origin and evolution in the Archean unless substrates equivalent to dunites (composed of > 90 wt% olivine) were more common.

## 1 Introduction

Phosphorus is a key component of cell membranes, DNA, and ATP, which together facilitate energy transfer, heritability, and the activity of all life on Earth. Phosphite (P(III)) is a reduced form of phosphorus that is more soluble than phosphate (P(V)) (Herschy et al. 2018) and thus potentially more bioavailable in natural environments. Several microorganisms can use phosphite to generate energy (Ewens et al. 2021; Figueroa et al. 2018; Schink et al. 2002) and to obtain the phosphorus needed for growth and development (Martinez et al. 2012). As a result, phosphite constitutes a significant amount of the total phosphorus reservoir in several environments, including freshwater lakes (Pasek et al. 2014; Han et al. 2013), wastewater treatment plants (Sadeghi and Jackson 2024), a geothermal pool (Pech et al. 2009) and serpentinite muds in deep‐sea vents (Pasek et al. 2022). Concentrations in these habitats range from 0% to 60% of the total phosphorus reservoir. To our knowledge, phosphite in marine environments has not been quantified, but some of the world's most abundant phytoplankton—*Prochlorococcus*—have been found genomically capable of importing and assimilating phosphite in the Sargasso and Caribbean Seas (Martinez et al. 2012). Therefore, phosphite is likely present and bioavailable in several ocean regions.

Of these environments where phosphite is present, serpentinizing hydrothermal vents are interesting because they have been proposed as cradles for the origin and evolution of life on Earth (Martin et al. 2008; Lane 2002). As a result, any biologically available molecules produced there could have been used in some of life's earliest metabolisms. Phosphite is highly soluble and more reactive towards organic matter than phosphate, so it could have played an important role in prebiotic chemistry (Pasek et al. 2017). It may also have been abundant in the Archean (Pasek et al. 2017), but whether it served as an important source of phosphorus for early microbial communities remains an open question. Preliminary thermodynamic models simulating the geothermal water:rock reactions that occur when olivine is serpentinized predict that phosphate would be reduced to phosphite at a range of temperatures (0°C–300°C) provided the water:rock ratio is below 0.2 (equivalent to less than 0.2 kg of water per kilogram of rock, Pasek et al. 2022). For comparison, the subsurface temperatures of the modern Lost City Hydrothermal field have been estimated to be approximately 200°C ± 50°C (Seyfried Jr et al. 2015). These results have been corroborated by the presence of phosphite constituting 23%, 43%, and 60% of the total phosphorus reservoir in serpentinite rocks of the Klamath Mountains and in serpentinite muds at two of three seamounts at the Mariana Forearc (Pasek et al. 2022). Whether this reduced phosphorus is bioavailable for the resident microbial communities remains unknown.

Biological analyses have identified genes that enable microbial life to access phosphite from the environment. They include *ptxABC* and *ptdC*, which facilitate the uptake of phosphite into microbial cells, as well as *ptxD*, which facilitates the oxidation of phosphite in the cytoplasm, and *ptxE, ptdF, ptdG, ptdH*, and *ptdI*, which function alongside *ptdC* and *ptxD* to collect electrons from phosphite for chemolithotrophic growth (Simeonova et al. 2010). If present, these genes serve as a hallmark indicating whether the microbe can use phosphite either as a source of phosphorus (*ptxABCD*) for assimilatory phosphite oxidation (APO) or as an electron donor to support chemolithotrophic growth (*ptxDEptdCFGHI*) in a process termed dissimilatory phosphite oxidation (DPO—Simeonova et al. 2010).

Investigations into the bioavailability of serpentinization‐derived phosphite are important because phosphate, the most common form of phosphorus in the ocean today, may have been highly depleted in Precambrian seawater (Jones et al. 2015; Reinhard et al. 2017; Kipp and Stüeken 2017; Bjerrum and Canfield 2002; Planavsky et al. 2022). The lack of dissolved oxygen in the water column at that time would have suppressed remineralization of organic‐bound phosphate (Kipp and Stüeken 2017), while iron minerals trapped phosphate in sediments (Jones et al. 2015; Reinhard et al. 2017). Others have contested this view (Brady et al. 2022; Rasmussen et al. 2021; Ingalls et al. 2022), and so it remains an area of active debate (Rego et al. 2023).

Several natural processes can generate phosphite. These include lightning (Bindi et al. 2023), metamorphic reactions between phosphate and ferrous iron (Herschy et al. 2018; Baidya et al. 2024), serpentinization (Pasek et al. 2022), and the breakdown of schreibersite, an iron–phosphorus mineral delivered by meteorites (Pasek 2008). Of these, serpentinization, that is, a family of geochemical reactions between olivine or pyroxene minerals and liquid water (see examples in Schulte et al. 2006; Seyfried et al. 2007), is perhaps the most promising source of phosphite for life because ultramafic volcanic rocks (e.g., komatiites and peridotites) rich in these minerals were common on the Archean seafloor (Leong et al. 2021; Condie and O'Neill 2010), and serpentinization‐driven hydrothermal vents actively disperse the products of serpentinization into the water column. If phosphate was depleted, even temporarily or within individual basins, phosphite could have been an important source of phosphorus because it is more soluble than phosphate, so elevated phosphite inputs could have solved the “phosphorus crisis.”

Phosphite has been found in some Archean marine carbonates (Pasek et al. 2013), suggesting that phosphite was present in the ocean 3.52 billion years ago (Ga). The source of this phosphite is uncertain, but a subset of life has likely been accessing phosphite to support growth and development for billions of years. Phylogenomic analyses estimate that *ptxB*, a key binding protein for microbial uptake of phosphite, first emerged in the Neoarchean to Paleoproterozoic (Boden et al. 2024). Other methods of phosphite uptake exist (e.g., *ptdC*) and have not been dated, but *ptxD*, which is key for all microbial phosphite metabolisms, is estimated to have emerged around the same time, toward the end of the Archean and into the Proterozoic (Boden et al. 2024). Komatiites, which are olivine‐rich volcanic rocks that can undergo serpentinization during exposure to anoxic water, disappear from the geological record around 2.0 Ga (Condie and O'Neill 2010), and therefore either of these estimates for the antiquity of phosphite‐metabolizing enzymes could be compatible with a hydrothermal source.

Here, we investigate the inventory of phosphorus‐metabolizing genes in microbial communities around modern serpentinizing and non‐serpentinizing vents and springs to test whether phosphite is bioavailable for organisms around hydrothermal vents. We also provide insight into the hypothesis that serpentinizing hydrothermal vents supplied significant phosphite to Earth's early biosphere. Non‐serpentinizing sites were included for control. To further support the genomic analyses, we include thermodynamic modeling of phosphorus speciation during serpentinization that expands on previous work (Pasek et al. 2022) by incorporating pyroxenes as sources of cations (McCollom and Bach 2009), and hydroxyapatite as a sink for phosphate (Randolph‐Flagg et al. 2023). Together, these combined datasets suggest that few, if any, of the microbial communities at serpentinizing vent sites use phosphite for chemolithotrophic growth via DPO. Some can assimilate phosphite for phosphorus using *ptxABCD*, but others do not, potentially because of apatite saturation and because (according to our results) phosphate is more abundant than phosphite in vents hotter than 310°C with water:rock ratios higher than 0.2. We nonetheless identify a restricted chemical space in reaction temperature and rock composition where geothermal phosphite concentration exceeds that of phosphate within the compositional space of terrestrial ophiolitic and orogenic ultramafic rocks.

## 2 Materials & Methods

### 2.1 Geochemical Models

Numerical simulations of serpentinization reactions were accomplished using the EQ3/6 code, version 8.0a (Wolery and Jarek 2003; Wolery and Jove‐Colon 2004; Wolery 2013). EQ3/6 encompasses a speciation‐solubility code (EQ3NR) and a reaction path modeling code (EQ6). EQ6 solves for the co‐existing equilibrium in the fluid between gases, solid minerals, and aqueous species at a given temperature and pressure by using tabulated logK values supplied by a database and using modified Newton–Raphson methods to iterate until the solution converges (Nordstrom et al. 1979; Wolery and Jarek 2003). However, an explicit gas phase in the equilibrium system is not considered.

Results from geochemical modeling are limited to the accuracy and completeness of the thermodynamic data compiled in the database. We used the thermodynamic database “tde” of Randolph‐Flagg et al. (2023) using thermodynamic data assessed by Ely (2020), given its inclusion of phosphorus species. These include orthophosphate (${\mathrm{HPO}}_{4}^{2-}$, ${\mathrm{PO}}_{4}^{3-}$, $H_{2}{\mathrm{PO}}_{4}^{-}$, H_3_PO_4(aq)_), phosphites (${\mathrm{HPO}}_{3}^{2-}$, $H_{2}{\mathrm{PO}}_{3}^{-}$, H_3_PO_3(aq)_), pyrophosphates ($P_{2}O_{7}^{4-}$, ${\mathrm{HP}}_{2}O_{7}^{3-}$, $H_{2}P_{2}O_{7}^{2-}$, $H_{3}P_{2}O_{7}^{-}$, H_4_P_2_O_7(aq)_), and hypophosphites ($H_{2}{\mathrm{PO}}_{2}^{-}$, H_3_PO_2(aq)_). Included phosphate‐bearing minerals are calcium‐bearing hydroxyapatite (Ca_5_(PO_4_)_3_OH), chloroapatite (Ca_5_(PO_4_)_3_Cl), and fluorapatite (Ca_5_(PO_4_)3F). Crystalline apatite is used as a sink, noting that it can be kinetically inhibited from forming in natural environments when Mg reaches a threshold relative to Ca (Martens and Harriss 1970). An amorphous Ca‐P precipitate forms instead of a crystalline species. In time, however, apatite will form (Gulbrandsen et al. 1984). Therefore, allowing hydroxyapatite as a thermodynamic sink is consistent with natural processes removing phosphorus from solution. Such an efficient removal of phosphate from solution has been used to explain phosphate‐poor environments in calcium‐containing systems (Toner and Catling 2019).

The list of P‐species in the model is necessarily incomplete due to limited experimentally determined thermodynamic data for phosphorus‐bearing chemical species. Indeed, source phosphorus bound in olivine and sinks such as phosphate salts or mineral phases containing reduced phases cannot be explicitly modeled (but are considered—see below). Nevertheless, this list of available species in the thermodynamic database has been conditionally vetted. Indeed, employing this database with EQ3/6, Randolph‐Flagg et al. (2023) successfully predicted the phosphate concentration detected in the plume of Saturn's moon Enceladus (Postberg et al. 2023). The phosphate detected on Enceladus, as modeled by Randolph‐Flagg et al. (2023) is released by analogous water:rock reactions to our study. The conditional statement is made because neither Randolph‐Flagg et al. (2023) nor Postberg et al. (2023) report explicit phosphite values. The temperature range of this database is 0°C–400°C and is set at a fixed pressure of 500 bars, corresponding to a water depth of 5 km on Earth. Thermodynamic properties of species (other than gasses) in serpentinization are relatively insensitive to pressure (McCollom and Bach 2009). Our computational peridotite is composed of the database solid solution minerals “IDEAL OLIVINE,” “CLINOPYROXENE,” and “ORTHOPYROXENE.” Antigorite is suppressed in favor of chrysotile (Evans et al. 2013). We also suppressed graphite, the Ca‐bearing silicate minerals monticellite, and andradite (Klein et al. 2013). Additionally, following Klein et al. (2013), we suppressed mineral carbonate formation (specifically calcite, dolomite, magnesite, aragonite, huntite, siderite, hydromagnesite, artinite and nesquehonite as they represent all the relevant carbonate minerals in the tde database). The formation of methane and methanol is also suppressed following the kinetic arguments of McCollom (2016). The thermodynamic database of Klein et al. (2013) was updated in McCollom et al. (2022). The two databases are compared in Figure S1 and return consistent normalized alteration mineralogy. The database of McCollom et al. (2022) does return higher amounts of high‐temperature magnetite (< 10% of the normalized assemblage) between 200°C and 325°C. Detailed comparative assessments are beyond the scope of this study but do invite future work. To what extent this small difference in alteration mineralogy impacts the formation of P‐bearing species at thermodynamic equilibrium is unclear. Therefore, we use the database of Randolph‐Flagg et al. (2023) as is.

The starting composition of the reacting fluid is shown in Table 1. The fluid represents terrestrial seawater devoid of oxygen and sulfate with 2 μM ${\mathrm{HPO}}_{4}^{-}$. This value was chosen because it represents the mean phosphate concentration of the modern ocean (Anderson and Sarmiento 1995). The lack of initial oxidants should provide a phosphite upper‐bound at equilibrium conditions, further driven by the lack of phosphite minerals in the thermodynamic database. Initial pH was set to 7.8 (that of seawater at depth—McCollom and Bach 2009), the redox constraint was set to Eh = 0.0 V, and electrical charge balance was on the most abundant ion, Cl^−^, so that any change in its concentration needed to maintain charge neutrality would affect minimally seawater composition. The simulated reacting rock was a peridotite of mineral composition defined by solid solutions (McCollom and Bach 2009; Klein et al. 2013), specifically N moles of olivine (N*0.1 fayalite, N*0.9 forsterite; Fo_90_), M moles of orthopyroxene (M*0.1 ferrosilite, M*0.9 enstatite′ En_90_), and Q moles of clinopyroxene (Q*0.1 hedenbergite, P*0.9 diopsite, Di_90_), where N, M, and Q were varied to cover the full ultramafic ternary diagram (Figure S1). Additionally, phosphorus is a known constituent of the upper‐mantle (Watson 1980). We use their estimate of ~200 ppm, equivalent to ~6.45 × 10^−3^ moles of phosphorus per kg of peridotite, in our simulations. Because one cannot add phosphorus to the predefined olivine solid solution listed in the database, we reflect this upper‐mantle P by additionally including 2.15 × 10^−3^ moles of fictive chloroapatite (Ca_5_(PO_4_)_3_Cl) as part of the rock composition to act as a source of P. The added other ionic species, order 10^−2^ mole Ca^++^ and order 10^−3^ mole Cl^−^ do not meaningfully add to their respective ion concentrations in the geochemical system. Indeed, reacting seawater contains order 10^−2^ mole Ca^++^ and order 10^−1^ mole Cl^−^ (Table 1). These abundances will thus minimally impact the final equilibrium result. Importantly, no chloroapatite remains at equilibrium, meaning that the calcium, chlorine, and phosphate from chloroapatite are fully dissolved by the water:rock reaction and distributed into the chemical system. This suggests chloroapatite is a valid fictive vehicle on which to deliver phosphate to the system in the absence of the ability to add phosphorus directly into the olivine composition. For this work, we updated the EQ3/6 wrapper code *chEQWRk* v0.1 of Som et al. 2024 and re‐released it here as *v0.2* (Som, 2025) for ease in calculating and reproducing the results. Released *chEQWRk* versions are available on GitHub (https://github.com/sanjoymsom/chEQWRk/).

**TABLE 1:** Starting composition of reacting fluid for geochemical models.

Starting seawater composition [units of all species except pH: Molality × 10^−3^]	
pH	7.8^a^
Na^+^	464
Cl^−^	546
${\mathrm{HCO}}_{3}^{-}$	2.3
${\mathrm{HPO}}_{4}^{-}$	2 × 10^−3^
Ca^2+^	10.2
Mg^2+^	24.8
K^+^	9.8
SiO_2(aq)_	0.16
Fe^2+^	1.5 × 10^−6^

*Note:* Seawater composition is taken from McCollom and Bach (2009), and represents seawater modified by crustal circulation: The fluid is devoid of oxygen and sulfate, with the concentrations of Ca and Mg diminished for charge balance.

^a^ pH = 7.8 is that of the ocean at depth. Surface water pH is 8.2.

### 2.2 Metagenomic Analyses

Metagenomic data representing the microbial communities at various serpentinizing and non‐serpentinizing sites were sourced from previously published papers (Tables 2 and 3). Some (namely those from Lost City Hydrothermal Field, the Coast Range Ophiolite Microbial Observatory (CROMO), The Cedars, Axial Seamount, and Axial Background) had already been trimmed and assembled (see the references in Tables 2 and 3), whereas others (namely those from the Mariana Forearc, Voltri Massif, and Prony Bay hydrothermal field (PHF)) were not. In these cases, reads were trimmed with Trimmomatic v. 0.39 (Bolger et al. 2014) in paired‐end mode using ‘‐phred33’ to read the quality scores, LEADING:20 to remove low‐quality bases from the start of each read, TRAILING:20 to cut low‐quality bases from the end of each read, SLIDINGWINDOW:4:20 to cut reads once the average quality within a 4‐base window falls below 20 and MINLEN:50 to remove short reads. Low quality is defined as anything with a phred score < 20 to disregard base calls with less than 99% accuracy. Similar trimming methods have been applied previously (Chrismas et al. 2016). ILLUMINACLIP was applied in Trimmomatic v. 0.39 (Bolger et al. 2014) to remove adapter sequences from reads of the Mariana Forearc, The Cedars, and Prony Bay because they were either sequenced using a Nextera DNA Flex library preparation kit (Mullis et al. 2023) or adapters were found with FastQC v0.12.1 (Andrews 2024). In contrast, ILLUMINACLIP was not used to remove adapters from reads of the Voltri Massif because they were sequenced using the Nugen Ultralow Ovation kit for library construction (Brazelton et al. 2017), and no adaptors were found in the FastQC reports. The resulting high‐quality reads were then decontaminated by removing reads that mapped onto the genomes of seven taxa known to contaminate DNA extraction kits with foreign genetic material that did not originate from the sample site (Mullis et al. 2023). This mapping was conducted with BBMap v.39.03 (Bushnell 2023) in ‘perfectmode’ to ensure only exact matches were removed. Finally, decontaminated reads were assembled de novo with SPAdes v3.15.5 (Bankevich et al. 2012) using default k‐mers of length 21, 33, and 55 and the ‘meta’ flag to account for microdiversity challenges associated with mixed microbial communities (Nurk et al. 2017).

**TABLE 2:** Source of metagenomes and metatranscriptomes from serpentinizing vents.

Name	Habitat	Source	Accession number(s)	Sample type	Number of samples	Assembly method	Type
Lost City Hydrothermal Field	Marine	Brazelton et al. (2022)	BioProject PRJNA779602 on the NCBI	Diffuse fluid	7	Pre‐trimmed and pre‐assembled	Metagenomes
Lost City Hydrothermal Field	Marine	Brazelton et al. (2022)	BioProject PRJNA779602 on the NCBI	Diffuse fluid	2	Pre‐trimmed and pre‐assembled	Metatranscriptomes
CROMO	Terrestrial	Sabuda et al. (2021)	BioProject PRJNA672823 on the NCBI	Well fluid	5	Pre‐trimmed and pre‐assembled	Metagenomes
Voltri Massif	Marine	Brazelton et al. (2017)	BioProject PRJNA265986 on the NCBI	Fluid	4	Raw reads were downloaded from NCBI SRA, then decontaminated and assembled using the methods of Mullis et al. (2023) (minus the removal of drill fluid control)	Metagenomes
Von Damm (at Mid Cayman Rise)	Marine	Anderson et al. (2017)	PRJEB15541 in the European Nucleotide Archive	Diffuse fluid	10	Pre‐trimmed, pre‐decontaminated and pre‐assembled	Metagenomes
The Cedars	Terrestrial	Suzuki et al. (2017, 2024)	BioProject PRJDB2971I on the NCBI	Fluid	5	Pre‐trimmed and pre‐assembled	Metagenomes
The Cedars	Terrestrial	Suzuki et al. (2017, 2024)	BioProject PRJDB2971I on the NCBI	Fluid	3	Pre‐trimmed and pre‐assembled	Metatranscriptomes
Prony Bay hydrothermal field	Marine	Mei et al. (2016)	SRR1636517 and SRR1636516 non the NCBI Short Read Archive	Chimney sections	2	Raw reads were downloaded from NCBI SRA, then decontaminated and assembled using the methods of Mullis et al. (2023) (minus the removal of drill fluid control)	Metagenomes
Old City	Marine	Lecouvre et al. (2020)	BioProject PRJNA556392 on the NCBI	Chimney sections	M3	Raw reads were downloaded from NCBI SRA, then decontaminated and assembled using the methods of Mullis et al. (2023) (minus the removal of drill fluid control)	Metagenome
Mariana Forearc	Marine	Mullis et al. (2023)	BioProject PRJNA592129 on the NCBI	Mud	8	Raw reads were downloaded from NCBI's SRA, then decontaminated using the methods of Mullis et al. 2023 and assembled de novo with SPAdes v3.15.5	Metatranscriptomes

**TABLE 3:** Source of metagenomes from non‐serpentinizing vents and background seawater for Axial Seamount.

Name	Habitat	Source	Accession number(s)	Sample type	Number of samples	Assembly method	Type
Piccard vent field (at Mid Cayman Rise)	Marine	Anderson et al. (2017)	PRJEB15541 in the European Nucleotide Archive	Diffuse fluid	4	Pre‐trimmed, pre‐decontaminated and pre‐assembled	Metagenomes
Axial Seamount	Marine	Fortunato et al. (2018)	Submission numbers 74596, 74599, 74602, 71132, 71134, 71135, 78375, 78377, 78401 & 78372 on IMG/MER	Diffuse fluid	14	Pre‐trimmed, pre‐decontaminated and pre‐assembled	Metagenomes
Axial Background	Marine	Fortunato et al. (2018)	Submission number 78368 on IMG/MER	Fluid	1	Pre‐trimmed, pre‐decontaminated and pre‐assembled	Metagenome

To find out whether microbial communities can use reduced phosphorus species at serpentinizing vents, the resulting metagenomes and metatranscriptomes were interrogated for homologs of genes that import, oxidize, or catabolize phosphite (${\mathrm{HPO}}_{3}^{-}$), phosphonates (PO_3_R) and phosphate (${\mathrm{PO}}_{4}^{3-}$, $H_{2}{\mathrm{PO}}_{4}^{-}$) (Table 4). Assemblies from all the vents at a given site were concatenated and searched for homologs of the enzymes listed in table 4 with tblastn (Altschul et al. 1990) using diverse query sequences (Tables S1 and S2) and an e‐value cut‐off of < 1 × 10^−25^ to reduce the possibility of finding hits by chance as opposed to genuine homology. Hits that aligned to < 70% of the mean length of the query sequences for the targeted genes were removed; then further stringency criteria were applied to remove spurious sequences based on relatedness. These involved aligning all the homologs of a given protein with the query sequences used to identify them using default options in mafft v7.453 (Katoh and Standley 2013) and reconstructing their evolutionary history using fasttree v. 2.1.11 (Price et al. 2010), also with default parameters. The resulting phylogenies were rooted using minimal ancestor deviation (Tria et al. 2017) because minimal ancestor deviation has been found to be one of the two most accurate methods of rooting prokaryotic gene families (Wade et al. 2020). The resulting rooted trees were visualized in TreeViewer v. 2.2.0 (Bianchini and Sánchez‐Baracaldo 2024) so that distant homologs that did not descend from the most recent common ancestor (MRCA) of the query sequences were removed. By filtering the tblastn hits in this way, we took a careful approach to gene identification by assuming that evolutionarily distant homologs of known phosphorus‐cycling genes may not have the same biological function. For *ptdC*, an additional sequence encoding PtdC in *Phosphitivorax anaerolimi* Phox21 (NCBI ID HPW69547.1; Figueroa et al. 2018) was added to supplement the single query sequence used in tblastn. Homologs of *palB* and *ppd* were only considered present if they were at a similar locus to *pepM* in the same contig.

**TABLE 4:** Genes involved in the microbial utilization of reduced phosphorus compounds and phosphate.

Phosphorus compound	Gene	Function
Phosphite	*ptxA*	Encodes the ATPase component of an ABC transporter which imports phosphite
	*ptxB*	Encodes the periplasmic binding protein of an ABC transporter which imports phosphite
	*ptxC*	Encodes the permease of an ABC transporter which imports phosphite
	*ptxD*	Encodes phosphite dehydrogenase which oxidizes phosphite to phosphate
	*ptxE*	Part of the dissimilatory phosphite oxidation operon
	*ptdC*	Encodes a symporter which imports phosphite in exchange for exporting phosphate
	*ptdF*	Part of the dissimilatory phosphite oxidation operon
	*ptdG*	Part of the dissimilatory phosphite oxidation operon
	*ptdH*	Part of the dissimilatory phosphite oxidation operon
	*ptdI*	Part of the dissimilatory phosphite oxidation operon
Phosphonate	*phnJ*	Encodes an enzyme of the C‐P lyase complex which cleaves the C‐P bond
	*phnM*	Encodes an enzyme of the C‐P lyase complex which creates a product for PhnJ to act upon
	*phnZ*	Encodes a phosphonohydrolase which cleaves 1‐hydroxy‐2‐AEPn to release phosphate and glycine
	*phnX*	Encodes phosphatase which releases phosphate and acetaldehyde from phosphonoacetaldehyde
	*phnA*	Encodes phosphonoacetate hydrolase which releases phosphate and acetate from phosphonoacetate
	*phnW*	Encodes 2‐AEP aminotransferase which catalyzes a reversible reaction which converts 2‐AEPn to phosphonoacetaldehyde
	*pdh*	Encodes phosphonoacetaldehyde dehydrogenase which catalyzes a reversible reaction which converts phosphonoacetaldehyde to 2‐hydroxyethylphosphonate
	*pepM*	Encodes phosphoenolpyruvate mutase which catalyzes a reversible reaction to convert phosphoenolpyruvate to 3‐phosphonopyruvate and vice versa
	*ppd*	Encodes phosphonopyruvate decarboxylase which converts phosphonopyruvate into phosphonoacetaldehyde to push PepM in the direction of phosphonate production
	*palB*	Encodes phosphonopyruvate aminotransferase which converts phosphonopyruvate into phosphonoalanine to push PepM in the direction of phosphonate production
	*mpnS*	Encodes methylphosphonate synthase which converts 2‐hydroxyethylphosphonate into methylphosphonate
Phosphate	*pstS*	Encodes the substrate‐binding protein of a high affinity phosphate ABC importer
	*pnaS*	Encodes a low affinity symporter which imports phosphate (when available in high concentrations) in exchange for exporting Na^+^
	*pitH*	Encodes a low affinity phosphate transporter
	*pitB*	Encodes a low affinity phosphate transporter
	*pitA*	Encodes a permease which imports phosphate complexed to divalent cartoons such as Mg^2+^, Ca^2+^, Co^2+^, Mn^2+^ or Zn^2+^

Coverage of the filtered homologs was calculated by mapping the trimmed and decontaminated reads (including paired reads and singletons where available) from each vent onto assemblies. The total number of trimmed and decontaminated reads in each sample varied from 10,514,637 at ST09 Chimney P27 in PHF to 713,562,756 at Old Man Tree in Von Damm (Figure S2). For Lost City (Brazelton et al. 2022), CROMO (Sabuda et al. 2021) and Axial Seamount (Fortunato et al. 2018), these assemblies are co‐assemblies of all reads from all sites sampled at the hydrothermal field. Whereas for Von Damm (Anderson et al. 2017), Piccard (Anderson et al. 2017), Voltri Massif (Brazelton et al. 2017), and the Mariana Forearc (Mullis et al. 2023), separate assemblies were made from reads from each sample, and contigs from the same vent fields were concatenated together. Mapping was conducted using bowtie2 v. 2.4.1 with default settings (Langmead and Salzberg 2012). The resulting .sam file was converted to .bam using SAMtools view, sorted using SAMtools sort, and indexed using SAMtools index with default parameters (Danecek et al. 2021). Per base coverage was then estimated using SAMtools bedcov (Danecek et al. 2021) and converted to per gene coverage by dividing by gene length. To facilitate comparisons between samples, per gene coverage was normalized by dividing by the total number of mapped reads in the sample (calculated with SAMtools view ‐c). They were then multiplied by 10^9^ to produce a measure of coverage that can be interpreted as a proportional unit; reads per billion.

### 2.3 Taxonomic Classification of Contigs Containing Phosphite Utilizing Genes

Metagenomic contigs predicted to encode genes for phosphite uptake and assimilation were classified according to the Genome Taxonomy Database v220 (Parks et al. 2022) using the taxonomy module (‐‐lca‐mode 2) within MMseqs2 v.15.6 (Mirdita et al. 2021).

### 2.4 Influence of Background Seawater

Regression models of the normalized coverages of *ptxA, ptxB, ptxC*, and *ptxD* in diffuse fluid samples from Lost City, Axial Seamount, and Von Damm were fitted to magnesium concentrations using the ‘lm’ function of the ‘stats’ package in R v. 4.2 (R Core Team, 2022) based on four degrees of freedom and six samples.

## 3 Results

### 3.1 Heterogeneous Bioavailability of Phosphite in Serpentinizing Systems

**FIGURE 1:**
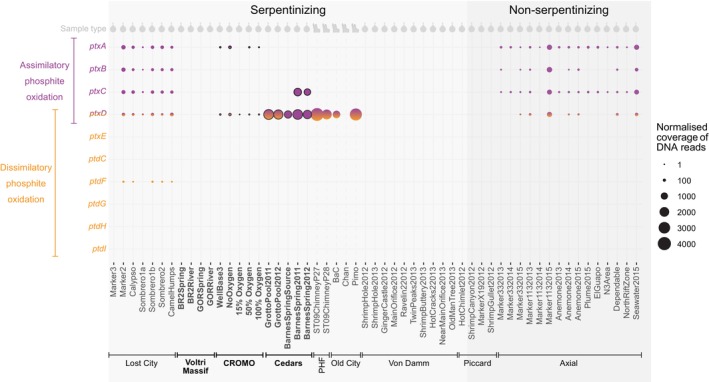
Abundance of genes for assimilatory (*ptxA, ptxB, ptxC* and *ptxD*) and dissimilatory (*ptxD, ptxE*, *ptdI, ptdH, ptdG, ptdF*, and *ptdC*) phosphite oxidation in microbial communities at serpentinizing (light grey) and non‐serpentinizing (dark grey) vents. Terrestrial hydrothermal fields are highlighted in bold text with black outlines. All others are marine. Grey symbols at the top of the graph indicate whether the samples were obtained from fluids (droplet symbols) or crushed chimney samples (chimney symbol). PHF, Prony bay hydrothermal field.

Microbial communities at five of the seven serpentinizing systems are genomically capable of oxidizing phosphite using *ptxD* (Figure 1). These include three of four marine and two of three terrestrial hydrothermal fields, namely chimney samples from PHF and Old City, as well as fluids sampled at Lost City, The Cedars, and CROMO (Figure 1). Of these, fluids from The Cedars and chimney samples from PHF and Old City host the highest normalized coverages of *ptxD* for phosphite oxidation (Figure 1, Table S3). The highest at each of these sites is at least 17 times larger than the highest measured in any other serpentinizing system (namely diffuse fluid from Camel Humps at Lost City, Figure 1, Table S3).

Despite its smaller relative abundance of phosphite‐oxidising genes, Lost City is the only serpentinizing field in our dataset that hosts microbial communities that are genomically capable of importing phosphite using an ABC transporter encoded by *ptxABC* (Figure 1). All three *ptxA*, *ptxB*, and *ptxC* genes are needed to successfully import phosphite (Metcalf and Wolfe 1998; White and Metcalf 2007), but CROMO contigs contain just the ATP‐binding protein encoded by *ptxA* (Figure 1). Similarly to CROMO, contigs from PHF and Old City lack *ptxA*, *ptxB*, and *ptxC*, and only two of five samples from The Cedars contain *ptxC* (Figure 1). Other methods of importing phosphite are known, such as *ptdC*, which encodes a phosphite/phosphate antiporter associated with DPO (Simeonova et al. 2010), but this was not present in CROMO or any of the hydrothermal vents tested. Only one of the seven genes associated with DPO (namely *ptdF*) was found in any of the hydrothermal fields, and this was found at just one hydrothermal field (namely Lost City). This gene is homologous to the *adpA* gene, which catalyzes a reaction between phosphite and adenosine monophosphate to produce adenosine diphosphate (ADP). ADP can be used for microbial growth, so the presence of *ptdF* could indicate an additional method of phosphite utilization by Lost City microbes (Mao et al. 2023). However, a full complement of genes, including *ptxE, ptdH*, and *ptdI*, as well as *ptdC* and *ptxD*, is required for DPO (Figueroa et al. 2018). Some of these DPO genes may be present in low abundances at Voltri Massif, PHF, and Old City because our conclusions are drawn from samples containing fewer than 40 million DNA reads (Figure S2) which may not be enough to capture low abundance genes (Kim et al. 2021). Therefore, our findings indicate a lack of DPO metabolism in most of the hydrothermal vents studied; but there is evidence to suggest that phosphite is bioavailable as a phosphorus source to microbes living in Lost City and potentially CROMO, The Cedars, PHF, and Old City via APO (Figure 1).

To test whether genes for microbial phosphite oxidation in diffuse fluid samples from Lost City originated from background seawater or from serpentinization, estimates of magnesium concentrations—a conservative tracer of seawater contamination (Fortunato et al. 2018)—were compiled. These were plotted against the normalized coverages of *ptxA, ptxB, ptxC*, and *ptxD* to investigate whether background seawater influenced the potential for microbial phosphite assimilation (Figure S3). The higher the [Mg], the more background seawater and the less hydrothermal fluid there is in the sample (Fortunato et al. 2018). Samples with higher [Mg] generally exhibited lower normalized coverages of *ptx* genes at Lost City, but there is no significant correlation between the degree of seawater contamination and normalized coverage of genes for microbial phosphite assimilation (*R* ^2^–0.3212, *p*‐value 0.4101).

In the absence of conclusive evidence from magnesium‐based tracers of seawater contamination, we classified contigs containing phosphite assimilation genes to test whether the phosphite‐oxidising genes were present in microbial species characteristic of hydrothermally altered areas or not. We were unable to classify contigs containing *ptxD* at PHF and Old City beyond Bacteria, but the contigs containing *ptxC* and *ptxD* at The Cedars were classified as *Roseococcus* and Dethiobacteraceae. Strains of the *Roseococcus* genus (Boldareva et al. 2009) and Dethiobacteraceae family have previously been isolated from soda lakes (Sorokin and Merkel 2022) confirming their association with terrestrial alkaline habitats. Similarly, the contig containing *ptxD* at CROMO was classified as Mycobacteriaceae, and the contig with *ptxA* was classified as *Dietzia natronolimnaea* A. This *Dietzia natronolimnaea* A isolate was first identified in an alkaline African soda lake (Duckworth et al. 1998). At Lost City, the contigs found to contain *ptxABCD* were classified as *Halopseudomonas* and two other Gammaproteobacteria (one belonging to the Burkholderiales order). Strains from all three of these clades are found in a wide variety of habitats, making it difficult to establish a source in background seawater or geothermally altered fluid. However, the contig containing *ptdF* at Lost City was classified as *JAJIG01 sp021775195* (from the Rhodospirillalles order), which has been observed in benthic marine environments (Genome Taxonomy Database 2022). Other contigs containing just one, two, or three of the *ptx* genes at Lost City were classified as *Vreelandella*, *Pseudothioglobus*, or the Flavobacterium *CAJIYG01 sp913063045*. *Vreelandella* have been found in a range of environments, including saline lakes, saline soils, ice, and seawater, but one strain was isolated from a deep‐sea hydrothermal vent (Zhu et al. 2025). Given these results, we hypothesize that at least some of the phosphite‐cycling genes at Lost City do not originate from background seawater.

**FIGURE 2:**
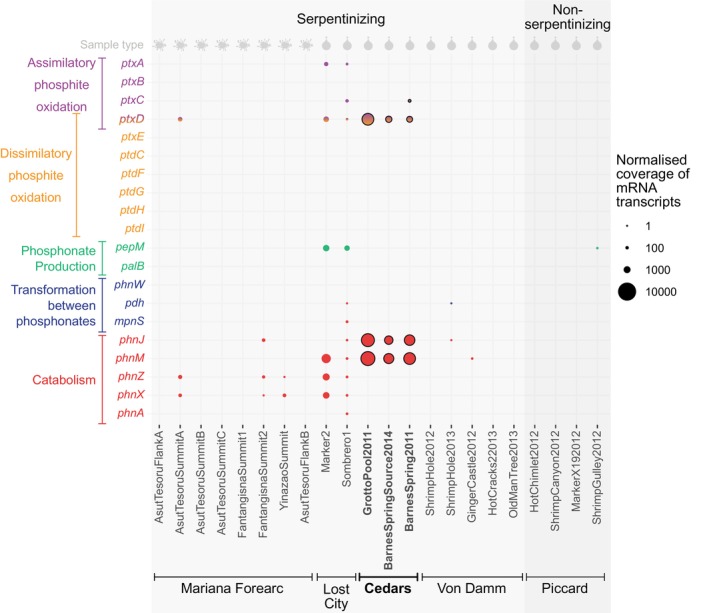
Active transcription of genes for reduced phosphorus metabolisms in microbial communities at serpentinizing (light grey) and non‐serpentinizing (dark grey) vents. Terrestrial hydrothermal vents are highlighted in bold text with black outlines. All others are marine. Grey symbols at the top of the graph indicate whether the samples were obtained from fluids (droplet symbol) or mud samples (mud splat symbol).

In addition to analyzing DNA sequences from the microbial community in the form of metagenomes, we also analyzed RNA sequences in the form of metatranscriptomes. This was done to investigate whether genes for phosphite uptake and oxidation were being actively used by microbial communities at Lost City, The Cedars, and the Mariana Forearc, which are all sites of serpentinization. We found that several phosphite uptake and oxidation genes present at Lost City (namely *ptxD, ptxA*, and sometimes *ptxC*) and The Cedars (namely *ptxD* and sometimes *ptxC*) were actively being transcribed by microbial communities in vents and pools where the data were available. At Lost City, this includes *ptxA, ptxC*, and *ptxD* at Sombrero 1 and *ptxA* and *ptxD* at Marker 2 (Figure 2). At The Cedars, *ptxD* and *ptxC* were transcribed by *Roseococcus* at Barnes Spring, and *ptxD* was transcribed by Bacillota of the Dethiobacteraceae family in Grotto Pool and Barnes Spring source water (Figure 2).

Further metatranscriptomes from sediment cores collected at the Mariana Forearc indicate active transcription of *ptxD*, which was classified as Rhizobium. This family is often associated with plants but has representatives that live in symbiotic relationships with marine planktonic diatoms (Tschitschko et al. 2024) and polychaete worms from benthic anoxic sediments (Summers et al. 2013). Perhaps surprisingly, *ptxD* transcription was only present at one of three Asùt Tesoru summit samples. No transcripts of *ptxA, ptxB, ptxC*, or *ptxD* were observed on the nearby flank or in samples from the summits of Fantangisa and Yinazao (Figure 2).

### 3.2 Bioavailable Phosphite in a Non‐Serpentinizing Hydrothermal Field

Six of 13 diffuse fluid samples from vents at Axial Seamount, one of the two non‐serpentinizing hydrothermal fields studied, hosted microbial communities capable of importing and oxidizing phosphite using *ptxA, ptxB, ptxC*, and *ptxD* (Figure 1). A full complement of all four genes was found in three of the seven sampled vents at Axial (namely Marker 113, Marker 33, and Anemone), but their presence varied over the 3 years studied, with *ptxD* being present in some years but absent in others (Figure 1). The contigs harboring all four of these genes were classified as Gammaproteobacteria or unclassified Pseudomonadota. Additional contigs containing one or two of the four *ptx* genes were classified as *Pseudothioglobus sp902606745* and *Marinobacter salarius* of the same Pseudomonadota phylum. *Marinobacter* species are widespread in marine environments that lack geothermal influence. For example, *Marinobacter salarius* has previously been isolated from shallow marine sediments in the Øresund Strait between the Baltic Sea and North Sea (Töpel et al. 2019) and coastal seawater samples at Chazhma Bay in the Sea of Japan (Ng et al. 2014).

The background seawater sample taken near Axial Seamount was relatively uninfluenced by geothermal activity and also contained Gammaproteobacteria and Pseudomonadota with these genes (namely *ptxA, ptxB, ptxC and ptxD*) (Figure 1). Given that seawater constituted 85%–96% of the diffuse fluid in all of the samples collected at Axial (Fortunato et al. 2018) and that similar species were present in both samples, the phosphite that is presumably driving the abundance of *ptxA, ptxB, ptxC*, and *ptxD* may originate from background seawater as opposed to geothermal activity.

### 3.3 A Potential Biological Phosphite Source at Axial Seamount, Lost City, CROMO, PHF, and Old City

**FIGURE 3:**
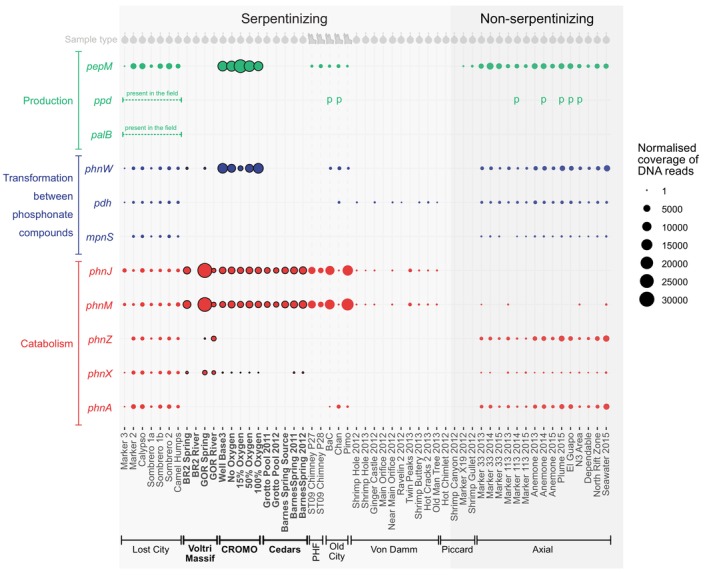
Abundance of genes for phosphonate production and catabolism in microbial communities at serpentinizing (light grey) and non‐serpentinizing (dark grey) vents. Terrestrial hydrothermal fields are highlighted in bold text with black outlines. All others are marine. The coverage of *ppd* and *palB* (for phosphonate production) cannot be calculated due to localization criteria, but their presence is indicated with a ‘p’. Where genes are known to be present at the hydrothermal field, but cannot be tested in individual samples (because all sample reads were combined before assembly) dotted lines are shown. Grey symbols at the top of the graph indicate whether the samples were obtained from fluids (droplet symbols) or crushed chimney samples (chimney symbol). PHF, Prony Bay hydrothermal field.

In the context of aquatic environments, phosphite production has been hypothesized to occur by degradation, rearrangement, or reduction of organic phosphonate compounds (Pasek et al. 2014). These compounds are produced biologically by the action of two enzymes: PEP mutase (encoded by *pepM*) and Ppd or PalB (White and Metcalf 2007; Li and Horsman 2022). Other enzymes such as FrbC and VlpB can also work with PepM to produce phosphonates, but they are much rarer, being present in only 8.1% of bacterial genomes as opposed to the 91.9% encoding Ppd or PalB (Li and Horsman 2022). At Axial Seamount, genes encoding PEP mutase and Ppd were present in microbial communities, indicating a potential biological source of reduced phosphorus (Figure 3). Similar genes for biological phosphonate production were also present in Lost City, CROMO, PHF, and Old City, where resident microbial communities are also biologically capable of oxidizing phosphite (Figure 3). Looking toward other sites where phosphite is not biologically available (namely the Voltri Massif, Von Damm, and Piccard), there is no evidence of *pepM, ppd*, or *palB* (Figure 3). Therefore, all microbial communities that are genomically capable of oxidizing phosphite are also genomically capable of producing organic phosphonates, except for those at The Cedars. This implies that at least some of the utilized phosphite at Lost City and potentially CROMO, PHF, Old City, and Axial Seamount could originate from catabolized phosphonates, in addition to, or instead of the phosphite originating from geothermal or seawater sources.

### 3.4 Phosphate Uptake

We estimated the coverage of genes encoding five orthophosphate importers to address how phosphite compares to orthophosphate as a source of phosphorus for microbial communities (orthophosphate describes the total amount of phosphate monomers as opposed to ‘phosphate’ which includes all chemical species containing a PO_4_‐group). We found evidence to suggest that microbes are genetically capable of importing orthophosphate bound to divalent metal ions in four vents at Von Damm, all six vents at Lost City, and one vent at Axial Seamount because *pitH* and/or *pitA* were present (Figures S4–S6). These genes import orthophosphate bound to Mg^2+^, Ca^2+^, Co^2+^, Mn^2+^, or Zn^2+^ (Martín and Liras 2021). None of the communities collected orthophosphate using *pitB*—a specific homolog found in *E. coli* (Hoffer et al. 2001)—or *pnas*. This *pnas* gene is more prevalent in phosphate‐replete environments (Boden et al. 2024), so its absence implies that orthophosphate is depleted. In line with this reasoning, *pstS*, encoding the key substrate‐binding component of a high‐affinity orthophosphate uptake system that enables microbes to access orthophosphate in phosphate‐depleted environments, is ubiquitous among all of the hydrothermal vents regardless of the type of geothermal activity present (Figures S4–S13). Together, this suggests that microbial communities prefer to import orthophosphate using *pstS* instead of *pnas* at all sites and that some orthophosphate is bound up with metal cations in Lost City and Von Damm.

**FIGURE 4:**
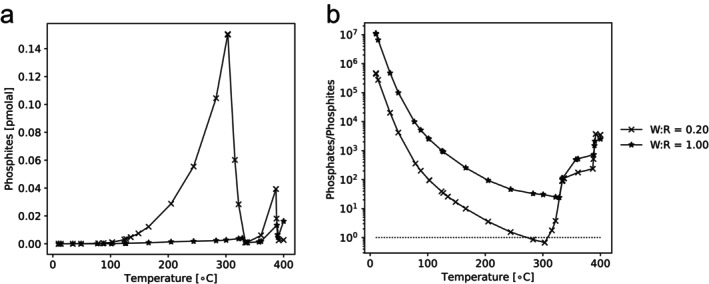
Estimated phosphite production ([${\mathrm{HPO}}_{3}^{-2}$] + [$H_{2}{\mathrm{PO}}_{3}^{-}$] + [H_3_PO_3(aq)_]) (a) and the ratio of orthophosphate ([${\mathrm{HPO}}_{4}^{-2}$] + [${\mathrm{PO}}_{4}^{-3}$] + [$H_{2}{\mathrm{PO}}_{4}^{-}$] + [H_3_PO_4(aq)_]) to phosphite production (b) in serpentinization reactions at different temperatures and water:Rock ratios. The dotted line in panel b indicates the phosphate:Phosphite ratio of 1. Below this, more phosphite is produced than orthophosphate. Numerical data underlying this figure is available in Data S1.

**FIGURE 5:**
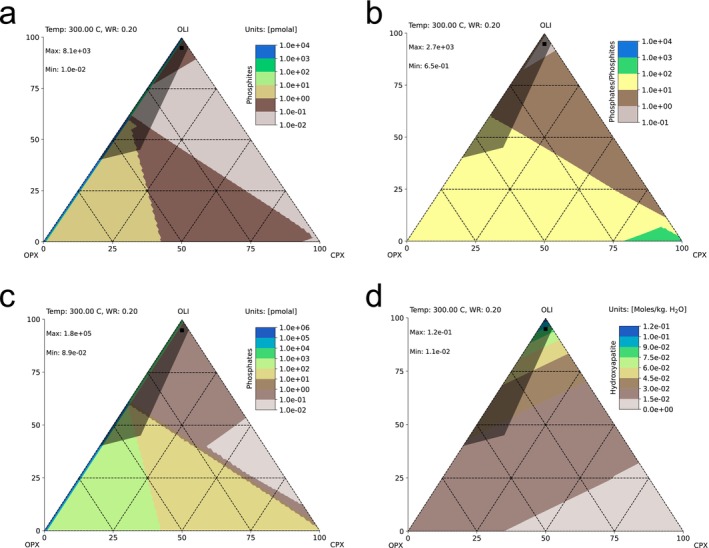
Ternary diagrams representing estimated phosphite production (a), the ratio of orthophosphate:Phosphite (b), absolute orthophosphate concentration (c) and absolute hydroxyapatite concentrations (d) in ultramafic serpentinites with different compositions of olivine (oli), clinopyroxene (cpx) and orthopyroxene (opx). Black squares at the top indicate the serpentinite composition used to predict phosphite and phosphate production with respect to temperature (Figure 5). Shaded regions correspond to the compositional space of terrestrial ophiolitic and orogenic ultramafic rocks (Ely et al. 2023). The diagram compiles 5130 individual simulations. Numerical data underlying this figure is available in Data S2.

The difference in the abundances of genes for orthophosphate and phosphite uptake mechanisms can provide insight into the preferences of microbial communities for reduced and oxidized phosphorus compounds. Most of the fluid samples (including those from CROMO, Barnes Spring at The Cedars and five of seven samples at Lost City) exhibited higher coverages of *pstS* than *ptxD* (Figures S6–S8), indicating that larger proportions of the resident bacterial and archaeal life maintain the genomic machinery to import orthophosphate than to oxidize phosphite. Such differences may reflect the serpentinization activity, which is expected to produce more phosphate than phosphite in most lithologies and temperature regimes (Figures 4 and 5). Alternatively, varying additional supplies of phosphate (and potentially phosphite; Van Mooy et al. 2015) could be produced as a result of the death and decay of microbial communities.

However, the differences between the coverages of genes for orthophosphate uptake and phosphite assimilation are smaller in diffuse fluids from Calypso and Marker 2 at Lost City (the only hydrothermal field hosting *ptxABC* for phosphite import as well as *ptxD* for oxidation) than those at CROMO (Figures S6 and S9). Phosphite also seems to be a relatively important source of phosphorus in fluid samples from Grotto Pool at The Cedars and chimney samples from PHF and Pimo at Old City where *ptxD* was present in higher normalized coverages than *pstS* (Figures S8, S12 and S13). Therefore, compared to phosphate, phosphite may represent a biologically more important source of phosphorus in chimneys at PHF and Old City and diffuse fluids from Grotto Pool at The Cedars compared to the other serpentinizing and non‐serpentinizing hydrothermal fields.

### 3.5 Geochemical Modelling

Our geochemical models estimate that phosphite concentrations in serpentinizing systems increase steadily from 0°C to 300°C at a water:rock ratio of 0.2, followed by a steep drop‐off and small spike in phosphite production at ~375°C (Figure 4a). In contrast, at the higher water:rock ratio of 1, which produces lower maximal absolute phosphite concentrations, the phosphite concentration is highest at 400°C (Figure 4a). This suggests that the temperature for maximum phosphite production varies depending on the relative proportions of water and rock in the serpentinite but is often at ~300°C or higher (Figure 4a). Within these trends, the concentration of phosphite remains low, at less than 10^−12^ M.

The amount of phosphite produced by serpentinization influences how much is available for the biological community. How much the microbes use could also depend on the availability of orthophosphate, which is also soluble. Genes for orthophosphate uptake are present at higher normalized coverages than those for phosphite uptake and oxidation at most of the hydrothermal vents studied, so if orthophosphate is abundant, it may be preferred (Figures S3–S9). Modeling predicts that the highest relative concentrations of orthophosphate to phosphite are produced via serpentinization at low temperatures approaching 0°C (Figure 4b). The orthophosphate:phosphite ratio decreases steadily with increasing temperatures until ~275°C at water:rock = 0.2 or 325°C at water:rock = 1 (Figure 4b). Above these temperatures, orthophosphate:phosphite ratios increase again (Figure 4b). Therefore, high‐temperature serpentinization reactions with low water:rock ratios offer the most potential for phosphite production and low orthophosphate:phosphite ratios (Figure 4).

To investigate the temperature effects just described, we chose a peridotite composition estimated to produce minimal orthophosphate:phosphite ratios (Figure 4b). Even in this optimal composition, orthophosphate:phosphite is always above 10 at water:rock = 1, representing 10 to 10 million times more orthophosphate than phosphite within the temperature range of 0°C–400°C (Figure 4b). The geochemical system only favors phosphite instead of orthophosphate production (indicated by orthophosphate:phosphite less than 1) at water:rock = 0.2 and a restricted temperature range (Figure 4b). As a result, consistently high phosphite productivity in relation to orthophosphate would require a relatively stable system with little variation in temperature and water influx.

In the geochemical space where phosphite is dominant over orthophosphate (T = 300°C, water:rock = 0.2), the protolith's starting composition ultimately affects the orthophosphate:phosphite ratio (Figure 5b). Absolute concentrations of phosphite vary from 0.01 to 8100 pmolal depending on the composition of the peridotite (Figure 5a). Low proportions of clinopyroxene—specifically less than 5%—combined with varying mixtures of orthopyroxene and olivine result in the highest absolute concentrations of 100–8100 pmolal phosphite (Figure 5a) due to low calcium abundance and thus minimizing apatite as a possible phosphorus sink. However, many of these compositions are estimated to produce more orthophosphate than phosphite (Figure 5b). As a result, more orthophosphate than phosphite is produced at all compositions except for a restricted compositional window of peridotite within that of terrestrial ophiolitic and orogenic ultramafic rocks, with similarly little clinopyroxene, but more than 87% olivine and less than 13% orthopyroxene (Figure 5b). Only at these compositions where orthophosphate:phosphite is between 1 and 0.1 are there equal or higher phosphite concentrations compared to orthophosphate at optimum temperature and water:rock ratio.

## 4 Discussion

### 4.1 Biological Constraints on Geothermal Phosphite Sources

Collectively, our biological and geochemical results may support previous findings indicating that geothermal phosphite can be produced via serpentinization (Pasek et al. 2022); however, this conclusion remains tentative. In support of a geothermal phosphite source, we find that the microbial community at Lost City is genomically capable of assimilating phosphite (using *ptxA*, *ptxB*, *ptxC* and *ptxD*), and bacterial communities at a further four serpentinizing sites in a range of marine and terrestrial settings are genomically capable of oxidizing phosphite (Figure 1). This expands on previous research which found *ptxD* for microbial phosphite oxidation in the serpentinizing system of Prony Bay (Frouin et al. 2022).

The lack of *ptxA, ptxB*, and *ptxC* for importing phosphite in several serpentinizing hydrothermal fields where sequencing was deep enough to capture low‐abundance genes (Figure S2) and where *ptxD* is prominent suggests that *ptxD* is useful in their absence. This may be because other methods of importing phosphite, such as the ATP transporter of the carbon‐phosphorus lyase operon (White and Metcalf, 2004; Metcalf and Wanner 1991) may be environmentally relevant and substitute for the action of *pxtA, ptxB*, and *ptxC*. In support of this, genes of the C‐P lyase operon were present in relatively high coverages in all sites where *ptxD* is present but *ptxA*, *ptxB*, and *ptxC* are missing (Figures 1 and 3).

Why microbial life at two other serpentinizing sites—namely the Voltri Massif and Von Damm—is not genomically capable of utilizing phosphite via *ptxA, ptxB, ptxC*, and *ptxD* remains unknown. They could be accessing phosphite using novel genes that have not been characterized yet. Alternatively, they could be accessing phosphite using carbon phosphorus lyases encoded by an operon containing *phnJ* and *phnM*, amongst others. Previous studies have found that *Pseudomonas stutzeri* and *Escherichia coli* can use this operon to grow on phosphite, albeit at lower rates than with *ptxABCD* (White and Metcalf 2004; Metcalf and Wanner 1991). These genes, *phnJ* and *phnM*, are ubiquitous and characteristic of the organisms around serpentinizing vents (Frouin et al. 2022). They are also present in higher normalized coverages than *ptxA*, *ptxB*, *ptxC*, and *ptxD* in 32 of the 35 serpentinizing vent samples in our dataset (Table S3, Figures 2 and 3). Therefore, phosphite may support life in all serpentinizing vents via carbon phosphorus lyases, but some sites, including Lost City and potentially CROMO, PHF, The Cedars, Old City, the Mariana Forearc (Figures 1 and 2), and Prony Island (Frouin et al. 2022) host microbial communities that are more efficient at using phosphite than others because they have *ptxD* as well as *phnJ* and *phnM*. This more efficient microbial oxidation of phosphite at Lost City, PHF, Old City, The Cedars, and CROMO could be a result of differing geochemistry of the underlying serpentinites, namely lower water:rock ratios, temperatures favoring geothermal phosphite production, lower phosphate concentrations, and/or larger exogenous inputs of phosphite from non‐geothermal sources.

However, it is also possible that the phosphite users at most of the serpentinizing sites are using phosphite from non‐geothermal sources, including background seawater and phosphite produced by the breakdown of phosphonates biosynthesized by microbial communities. If so, then our results would not support the proposition that serpentinization is an important source of phosphite for the biosphere. PEP mutase is the key enzyme needed for microbes to biosynthesize phosphonates. We find the *pepM* gene which encodes this enzyme, in several samples from Lost City, CROMO, Old City, PHF, the Mariana Forearc, and Axial Seamount. At these locations, phosphite must also be present because microbes have the most efficient genes for importing and oxidizing it (Figures 1 and 3). In contrast, *pepM* is correspondingly absent from all sites lacking *ptxA, ptxB, ptxC*, and *ptxD* for efficient phosphite assimilation (Figures 1 and 3), raising the possibility that phosphite could be both produced and oxidized by microbial activity around hydrothermal vents. Further study is required to find out how quickly phosphonates break down into phosphite under biologically relevant conditions, but it may occur inside microbial cells because phosphite has been found alongside methylphosphonate in *Trichodesmium* colonies (Van Mooy et al. 2015). Overall, our findings suggest that in marine systems, phosphite in diffuse fluids could originate from a mixture of geothermal and non‐geothermal processes.

Few measurements of phosphite and phosphate concentrations have been made in hydrothermal vents, but phosphite has been found to constitute 48% of the extractable phosphorus pool in serpentinite muds from Asut Tesoru at the Mariana Forearc (Pasek et al. 2022). This is the same vent in which we found active transcription of *ptxD* to oxidize phosphite in the microbial community (Figure 2), so the phosphite these organisms are using could originate from a geothermal source. Phosphite constitutes relatively less of the total phosphorus pool in other vents of the Mariana Forearc (Pasek et al. 2022), which lack transcripts of genes for phosphite uptake or oxidation (Figure 2). As a result, phosphite may only become biologically relevant when it represents a large proportion of the total phosphorus pool.

### 4.2 Geochemical Constraints on Geothermal Phosphite Production

In serpentinization‐driven hydrothermal fields where normalized coverages of the most efficient genes for oxidizing phosphite (White and Metcalf 2004) are small or absent compared to *pstS* for importing orthophosphate, microbial communities most likely prefer orthophosphate in comparison to phosphite as a phosphorus source. This was observed at CROMO, Voltri Massif, Von Damm, Barnes Spring at The Cedars, Chan chimney at Old City, and some of the vents at Lost City (namely Marker 3, Camel Humps, Sombrero1 and Sombrero2) (Figures S5–S8, S11 and S13). This raises the question of what limits phosphite production in these serpentinizing environments to render it biologically less useful compared to sites where coverages of the phosphite oxidizing gene (*ptxD*) were relatively higher (namely Lost City, PHF, Old City and Grotto Pool at The Cedars). Serpentinizing vents are among the most reducing environments on Earth. They generate high levels of H_2_ gas and contain metal catalysts capable of driving abiotic reduction of CO_2_ and N_2_ (Früh‐Green et al. 2004; Sleep et al. 2004). Previous detections of phosphite in serpentinite samples indicate that phosphate may undergo reduction in these settings (Pasek et al. 2022). However, our geochemical simulations suggest that the mobility of phosphate in serpentinizing systems is greatly limited by hydroxyapatite precipitation. Hence, if all initial phosphorus is present as orthophosphate (either derived from seawater or from the peridotite itself), apatite precipitation may limit the absolute amount of phosphite that can be generated.

Furthermore, orthophosphate is the more thermodynamically stable form of phosphorus under most conditions, except over restricted conditions at very low water:rock ratios and temperatures exceeding 275°C that nonetheless overlap with the expected compositional space of terrestrial ophiolitic and orogenic ultramafic rocks (Figure 4). Previous modeling studies suggest that the water:rock ratio in hydrothermal vents may be up to 5:1 (Wetzel and Shock 2000). Hence, most of the vent environments may not experience the conditions needed to maximize phosphate reduction. Furthermore, mixing with phosphate‐bearing seawater would further elevate the phosphate/phosphite ratio in vent effluents by dilution and possibly by abiotic oxidation with O_2_, which was omitted from our model to calculate an upper bound of phosphite production. Orthophosphate is the preferred phosphorus source for microbes because it requires fewer genes and, therefore, fewer nutritional resources to collect (White and Metcalf 2007), so dilution by phosphate‐bearing seawater may quickly reduce the biological utility of a small geothermal phosphite source.

We speculate that previous findings of phosphite in serpentinites (Pasek et al. 2022) and the relatively high coverages of *ptxD* for oxidizing phosphite in fluid from The Cedars and chimney samples (from PHF and Old City) may reflect end‐member conditions of low water:rock ratios occurring within minerals. At a mineral scale, it is possible to encounter pure olivine, which according to our results (Figure 5), would favor phosphate reduction. At the scale of the crust, the host rock of serpentinizing vents is composed of a mixture of olivine and orthopyroxene with minor clinopyroxene (Bodinier and Godard 2014, Ely et al. 2023). This would produce more phosphite than other compositions containing more calcium‐containing clinopyroxene (Figure 5), but phosphate still dominates under most water:rock conditions and temperatures. However, it is conceivable that secondary alteration of phosphite‐bearing serpentinites formed from pure olivine grains may liberate phosphite into the environment, and this flux could potentially contribute to phosphite metabolisms in some of our samples, especially in the chimney walls where organisms may be in direct contact with mineral grains such as olivine and its serpentinized derivatives. For example, previous analyses have observed mineral particles with a similar composition to olivine (composed primarily of silica, magnesium and iron) in fluid samples from The Cedars (Suzuki et al. 2017), but these have not been noted in fluids from other serpentinizing sites. These aggregates at The Cedars hosted the dominant microbial organisms (Suzuki et al. 2017). Perhaps in further support of this idea, Pasek et al. (2022) reported 50% phosphite in a sample from the Klamath Mountains with a harzburgite protolith that according to our model calculations should not have contained such a high fraction of phosphite. The phosphite found in that sample may have been imported from external, more olivine‐rich sources.

### 4.3 Implications for Early Life on Earth and Beyond

Hydrothermal activity was more common in the Archean (Viehmann et al. 2015), and serpentinizing vents, in particular, are likely to have been more common due to the prevalence of komatiite on the seafloor (Leong et al. 2021; Condie and O'Neill 2010). Therefore, if serpentinizing vents constituted a significant source of phosphite, i.e., a more soluble form of phosphorus than phosphate, they could have played a more important role in maintaining microbial communities and sustaining primary productivity. However, if geothermal phosphite is not available in sufficiently large quantities to support primary productivity in some modern serpentinizing hydrothermal fields (perhaps with the exception of local environments in direct contact with olivine grains), then it stands to reason that geothermally produced phosphite might only have been supporting life around a subset of vents in the past as well. This conclusion is further supported by molecular clock studies that have tracked the evolution of genes for biological phosphite utilization (namely *ptxB, ptxD*, *phnJ*, and *phnM*) to between the mid Mesoarchean boundary ~3 Ga and the end of the Great Oxygenation Event 2.32 Ga (Boden et al. 2024). This finding places a qualitative upper limit on phosphite sources to the biosphere in the middle of the Archean. If hydrothermal vents, either serpentinizing or non‐serpentinizing, had been a major phosphite source, an earlier origin of phosphite‐metabolizing genes would have been expected.

These analyses leave open the possibility that other phosphite‐metabolizing enzymes existed in the early Archean and have since become extinct. Importantly, serpentinization has been invoked to occur on icy moons, early Mars, and exoplanets (McCollom et al. 2022). Thermodynamic models of such extra‐terrestrial oceans suggest the possibility of elevated phosphate concentrations of > 10 μM (Randolph‐Flagg et al. 2023). High concentrations of up to 4100 μM have also been proposed for some environments on the early Earth (Brady et al. 2022). Under such phosphate‐rich conditions, the concentration of phosphite would also be elevated, following the phosphate/phosphite ratios calculated in our model (Figures 3 and 4). Since phosphite is more reactive towards organic matter than phosphate (Pasek et al. 2017), geothermal phosphite production may still have been important for prebiotic chemistry and the origin of life on Earth and other planets, even if its imprints are not archived in the evolutionary history of modern phosphite‐metabolizing genes.

## 5 Conclusion

Our metagenomic analyses and thermodynamic modeling suggest that phosphate is more thermodynamically stable than phosphite under most conditions of serpentinization. As a result, resident microbial communities in most fluids from serpentinizing sites (except two vents at Lost City and Grotto Pool in The Cedars) host higher normalized coverages of genes for phosphate compared to phosphite uptake from the environment. Although portions of the communities at Lost City, Old City, PHF, The Cedars, CROMO, and the Mariana Forearc can use phosphite (via *ptxD* and to a lesser extent via C‐P lyases), the presence of the *pepM* gene for biological phosphonate synthesis suggests that some of this phosphite could originate from the breakdown of biologically produced phosphonates as well as geothermal activity. Therefore, phosphite from serpentinizing vents is unlikely to have fueled substantial primary productivity in diffuse fluids during the Archean, unless low water:rock ratios and olivine‐rich substrates were more common. However, the phosphite from serpentinizing vents may have supported more notable primary productivity in benthic microbial communities inhabiting chimney walls and suspended olivine mineral particles.

## Conflicts of Interest

The authors declare no conflicts of interest.

## Supporting information


**Appendix S1.** Tables and figures.

**Data S1.** Numerical data underlying the geochemically‐modelled phosphite production at different temperatures and water:rock ratios in Figure 4.

**Data S2.** Numerical data underlying the geochemically‐modelled phosphite production in ultramafic serpentinites with different compositions of olivine, pyroxene and orthopyroxene in Figure 5.

## Data Availability

Code underlying geochemical models is available on github (https://github.com/sanjoymsom/chEQWRk/). Numerical results of the geochemical models are available in Data S1 and S2.
