# Supplementary material for: Evaluating Serpentinization as a Source of Phosphite to Microbial Communities in Hydrothermal Vents
Source: Geobiology. 2025 Mar 25;23(2):e70016. doi: 10.1111/gbi.70016 (PMC11933879; doi:10.1111/gbi.70016)

Supplementary Information for Manuscript Entitled: Evaluating Serpentinization as a Source of Phosphite to Microbial Communities

Table S1: Query sequences used to search for the genomic capability to use phosphite and hypophosphite molecules in microbial vent communities

Protein	Query Sequences		
	NCBI ID	Citation	Organism
PtxA	AAC71706.1	Metcalf & Wolfe, 1998	*Pseudomonas stutzeri* WM88
	WP_011863199.1	Martinez et al., 2012	*Prochlorococcus marinus* str. MIT 9301
	Q119J0.1	Polyviou et al, 2015	*Trichodesmium erythreaum* IMS101
PtxB	AAC71707.1	Metcalf & Wolfe, 1998<br>Bisson et al., 2017	*Pseudomonas stutzeri* WM88
	YP_001091475.1	Martinez et al., 2012<br>Bisson et al., 2017<br>Feingersch et al., 2012	*Prochlorococcus marinus* str. MIT 9301
	ABG49835	Bisson et al., 2017	*Trichodesmium erythreaum* IMS101
PtxC	AAC71708.1	Metcalf & Wolfe, 1998	*Pseudomonas stutzeri* WM88
	WP_011863201.1	Martinez et al., 2012	*Prochlorococcus marinus* str. MIT 9301
PtxD	YP_001091477.1	Martinez et al., 2012	*Prochlorococcus marinus str.* MIT 9301
	ADB92513.1	Simeonova et al., 2010	*Desulfotignum phospitoxidans*
	WP_003118429.1	Costas et al., 2001	*Pseudomonas stutzeri* WM88
	AAT12779.1	Wilson and Metcalf, 2005	*Alcaligenes faecalis* WM2072
PtxE	HPW69543.1	Figueroa et al., 2018	*Phosphitivorax anaerolimi* Phox21
	ADB92512.1	Simeonova et al., 2010	*Desulfotignum phosphitoxidans*
PtdC	ADB92515.1	Simeonova et al., 2010<br>Ewens et al., 2021	*Desulfotignum phosphitoxidans*
PtdF	HPW69548.1	Figueroa et al., 2018	*Phosphitivorax anaerolimi* Phox21
	ABU54327.1	Simeonova et al., 2010<br>Simeonova et al., 2009	*Desulfotignum phosphitoxidans*

PtdG	ADB92516.1	Simeonova et al., 2010	*Desulfotignum phosphitoxidans*
PtdH	HPW69546.1	Figueroa et al., 2018	*Phosphitivorax anaerolimi* Phox21
	ADB92517.1	Simeonova et al., 2010	*Desulfotignum phosphitoxidans*
PtdI	ADB92518.1	Simeonova et al., 2010	*Desulfotignum phosphitoxidans*
HtxA	AAC71711.1	Metcalf & Wolfe, 1998	*Pseudomonas stutzeri* WM88
HtxB	AAC71712.1	Metcalf & Wolfe, 1998	*Pseudomonas stutzeri* WM88
	AAT12776.1	Wilson and Metcalf, 2005	*Alcaligenes faecalis WM2072*

Table S2: Query sequences used to search for the genomic capability to use phosphonate and phosphate molecules in microbial vent communities

Protein	Query Sequences		
	ID	Database	Number of Seed Sequences
PhnJ	PF06007	Pfam domain	35
PhnM	TIGR02318.1	NCBI HMM	10
PhnZ	TIGR03276.1	NCBI HMM	10
PhnX	TIGR01422.1	NCBI HMM	11
PhnA	TIGR02335.1	NCBI HMM	3
PhnW	TIGR02326.1	NCBI HMM	5
Ppd	TIGR03297.1	NCBI HMM	10
PepM	TIGR02320.1	NCBI HMM	15
Pdh	TIGR03405.1	NCBI HMM	4
MpnS	WP_012214540.1	Sequence	1
PalB	PF00155	Pfam domain	47
PstS	TIGR00975.1	NCBI HMM	7
	NF008171.0	NCBI HMM	97
PNaS	NF037997.1	NCBI HMM	19
PitH	NBR010556	Blast Rule	2
PitB	WP_000933250.1	Sequence	1
PitA	NF033774.1	NCBI HMM	32

Table S3: Normalized coverages of *phnM* and *phnJ* in comparison to *ptxA*, *ptxB*, *ptxC* and *ptxD* in serpentinizing vent samples.

	phnJ	*phnM*	*ptxA*	*ptxB*	*ptxC*	*ptxD*
Marker 3	1245.79300	498.94800	0.00000	0.00000	0.00000	0.58700
Marker 2	272.36500	309.67100	264.20300	243.64800	209.17900	114.61600
Calypso	1506.73300	1452.50800	112.07900	68.60300	140.40600	58.04500
Sombrero 1a	508.93900	541.47700	8.57300	4.25700	11.82300	10.49600
Sombrero 1b	616.83100	761.21800	130.63700	81.71400	125.90300	100.05900
Sombrero 2	785.88600	625.09700	142.69700	86.20800	106.19000	37.99300
Camel Humps	818.72600	1396.72100	77.87900	94.40300	110.43600	142.07400
BR2 Spring	6916.83000	8554.72200	0.00000	0.00000	0.00000	0.00000
BR2 River	0.00000	0.00000	0.00000	0.00000	0.00000	0.00000
GOR Spring	29433.86200	28147.33600	0.00000	0.00000	0.00000	0.00000

	phnJ	*phnM*	*ptxA*	*ptxB*	*ptxC*	*ptxD*
GOR River	2187.04400	2594.98600	0.00000	0.00000	0.00000	0.00000
Well Base 3	5825.43500	4592.58000	27.13200	0.00000	0.00000	33.59900
No Oxygen	6186.46700	6539.64900	127.73800	0.00000	0.00000	105.16100
15% Oxygen	5140.94200	5034.77600	0.00000	0.00000	0.00000	4.21900
50% Oxygen	6053.70200	6403.35000	23.57700	0.00000	0.00000	14.89600
100% Oxygen	5378.23000	5428.96300	9.04500	0.00000	0.00000	16.81800
Grotto Pool 2011	4237.06700	4318.12100	0.00000	0.00000	0.00000	2515.37000
Grotto Pool 2012	3571.92400	3516.73400	0.00000	0.00000	0.00000	2197.17500
Barnes Spring Source	5607.72700	6205.88100	0.00000	0.00000	0.00000	1322.52900
Barnes Spring 2011	6440.59400	6704.75500	0.00000	0.00000	1421.74400	2410.16300
Barnes Spring 2012	7185.68300	7789.15100	0.00000	0.00000	1010.12300	1789.24500

	phnJ	*phnM*	*ptxA*	*ptxB*	*ptxC*	*ptxD*
ST09 Chimney P27	5324.42600	6759.22400	0.00000	0.00000	0.00000	3766.27600
ST09 Chimney P28	2706.01400	2825.07400	0.00000	0.00000	0.00000	2078.47700
BaC	9481.50500	11426.68400	0.00000	0.00000	0.00000	1086.40900
Chan	25.20200	31.66700	0.00000	0.00000	0.00000	0.00000
Pimo	12940.42000	18099.19700	0.00000	0.00000	0.00000	3240.58000
Shrimp Hole 2012	29.75200	28.07000	0.00000	0.00000	0.00000	0.00000
Shrimp Hole 2013	3.23800	11.81000	0.00000	0.00000	0.00000	0.00000
Ginger Castle 2012	29.83900	23.62900	0.00000	0.00000	0.00000	0.00000
Main Orifice 2012	0.00000	0.00000	0.00000	0.00000	0.00000	0.00000
Near Main Orifice 2013	39.71700	37.12300	0.00000	0.00000	0.00000	0.00000
Ravelin2 2012	0.00000	0.00000	0.00000	0.00000	0.00000	0.00000

	phnJ	*phnM*	*ptxA*	*ptxB*	*ptxC*	*ptxD*
Twin Peaks 2013	778.14600	731.19800	0.00000	0.00000	0.00000	0.00000
Shrimp Buttery 2013	58.64700	88.60500	0.00000	0.00000	0.00000	0.00000
Hot Cracks 2 2013	46.21800	67.82200	0.00000	0.00000	0.00000	0.00000
Old Man Tree 2013	53.75700	55.89400	0.00000	0.00000	0.00000	0.00000
Hot Chimlet 2012	0.00000	0.00000	0.00000	0.00000	0.00000	0.00000
Shrimp Canyon 2012	0.00000	0.00000	0.00000	0.00000	0.00000	0.00000
Marker X19 2012	0.00000	0.00000	0.00000	0.00000	0.00000	0.00000
Shrimp Gullet 2012	0.00000	0.00000	0.00000	0.00000	0.00000	0.00000
Marker 33 2013	0.00000	5.28700	51.40900	10.99000	11.51200	0.00000
Marker33 2014	0.00000	0.00000	39.85000	0.00000	26.22800	0.00000
Marker33 2015	0.00000	0.00000	7.92300	5.79600	13.24700	5.92000

	phnJ	*phnM*	*ptxA*	*ptxB*	*ptxC*	*ptxD*
Marker113 2013	0.00000	50.75000	42.91300	19.42800	36.47200	48.75500
Marker113 2014	0.00000	0.00000	2.19600	0.00000	0.00000	0.00000
Marker113 2015	0.00000	0.00000	436.22200	479.89500	372.89200	399.51100
Anemone 2013	0.00000	0.00000	41.87400	0.00000	22.68300	0.00000
Anemone 2014	0.00000	0.00000	47.40500	5.45000	28.05800	11.60900
Anemone 2015	0.00000	0.00000	39.82300	32.90500	26.46800	19.90800
El Guapo	0.00000	0.00000	64.67800	0.00000	33.98100	0.00000
N3 Area	0.00000	0.00000	8.44000	0.00000	10.73100	0.00000
Dependable	0.00000	62.33200	47.42400	24.97300	27.29000	61.60700
North Rift Zone	0.00000	0.00000	7.57000	0.00000	12.57400	0.00000
Plume 2015	0.00000	0.00000	71.19500	0.00000	40.71900	0.00000

	phnJ	*phnM*	*ptxA*	*ptxB*	*ptxC*	*ptxD*
Seawater 2015	0.00000	142.52400	403.08300	148.29100	345.39900	119.37200

Figure S1: Comparison of secondary mineral formation resulting from seawater reacting with harzburgite (95 wt% olivine, 2.5 wt% orthopyroxene 2.5 wt% clinopyroxene) using database from a) McCollom et al., 2022 ("mbn" database) and b) Randolph-Flagg et al., 2023 ("tde" database). McCollom et al. 2022 does not include the P-bearing species that Randolph-Flagg et al., 2023 have incorporated. For this reason, the latter was used in this study.

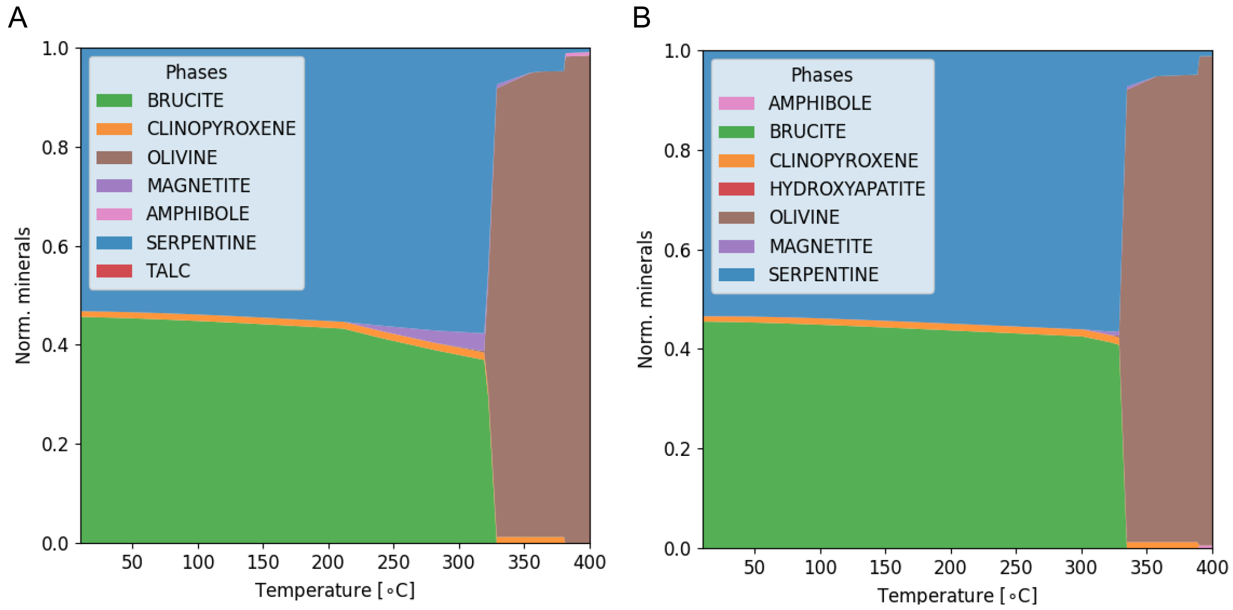

Figure S2: Total number of trimmed and decontaminated reads used to estimate the coverage of phosphorus-cycling genes in hydrothermal vent and background water samples. Samples containing less than the 40 million reads required to capture low abundance taxa (Kim et al., 2021) are highlighted in black.

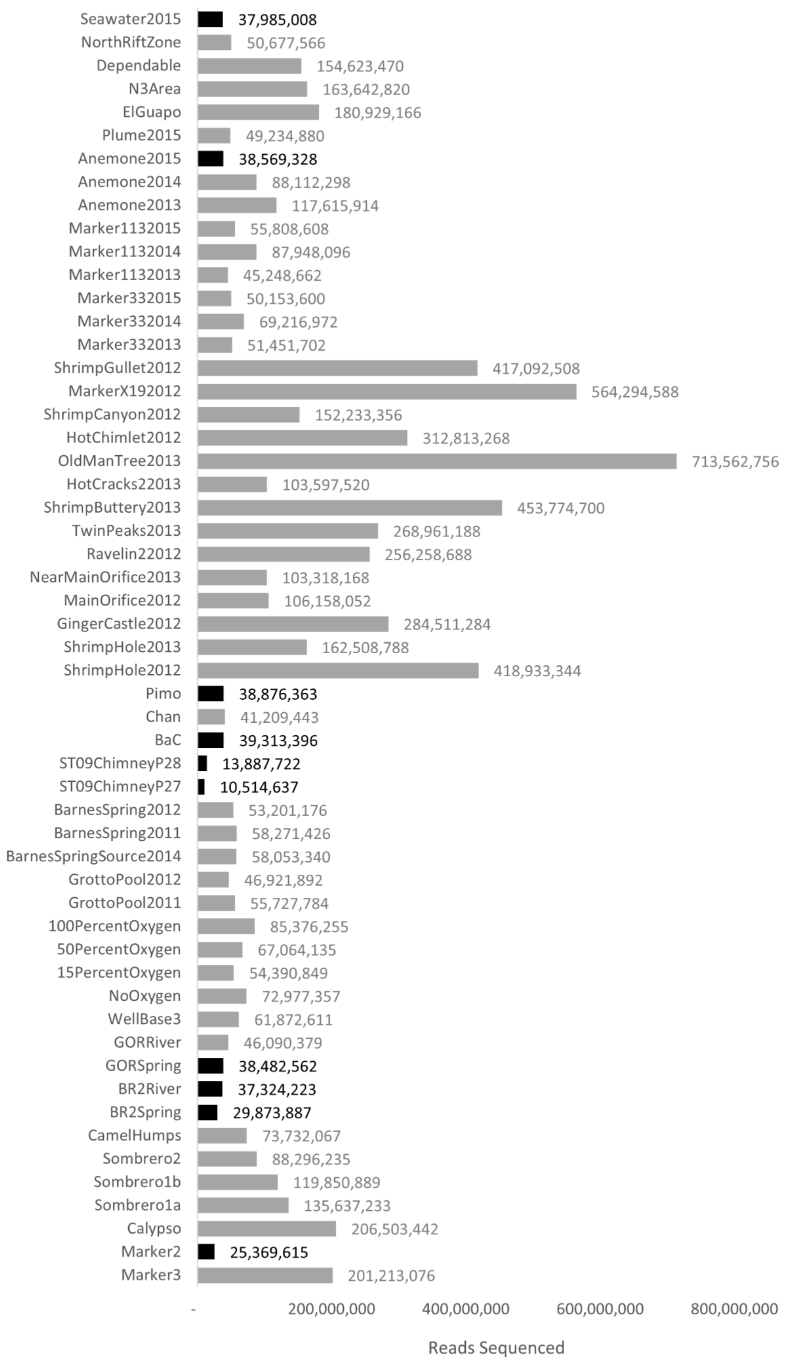

Figure S3: The degree of influence of background seawater (indicated by the conservative marker [Mg]) in diffuse fluid samples from marine hydrothermal vents and its relationship to the normalized coverages of *ptxD* (a), *ptxB* (b), *ptxC* (c) and *ptxA* (d). Magnesium concentrations in samples from Lost City (blue crosses), Von Damm (gray stars) and Axial Seamount (orange pluses) were sourced from previously published papers (Brazelton et al., 2022; Anderson et al., 2017 and Fortunato et al., 2018).

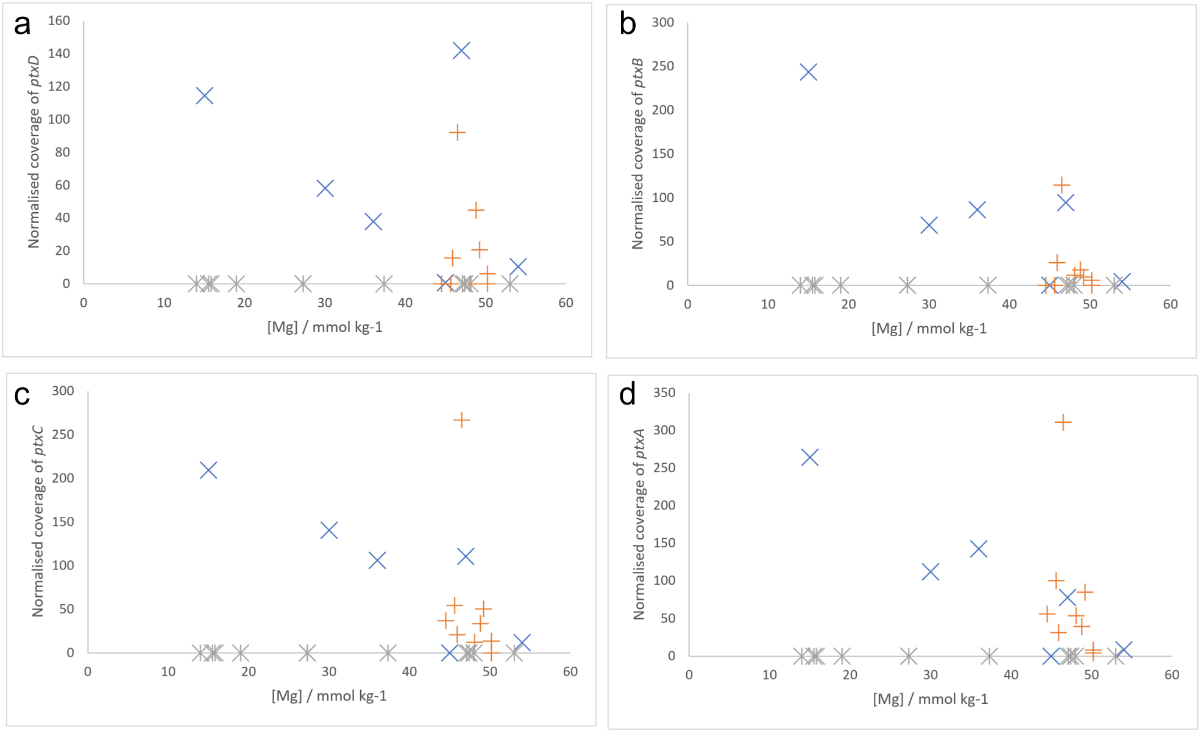

Figure S4: Relative coverage of genes for microbial phosphite utilization (blue; including import by *ptxA, ptxB* and *ptxC* as well as oxidation by *ptxD*) and phosphate uptake (yellow) at Axial Seamount.

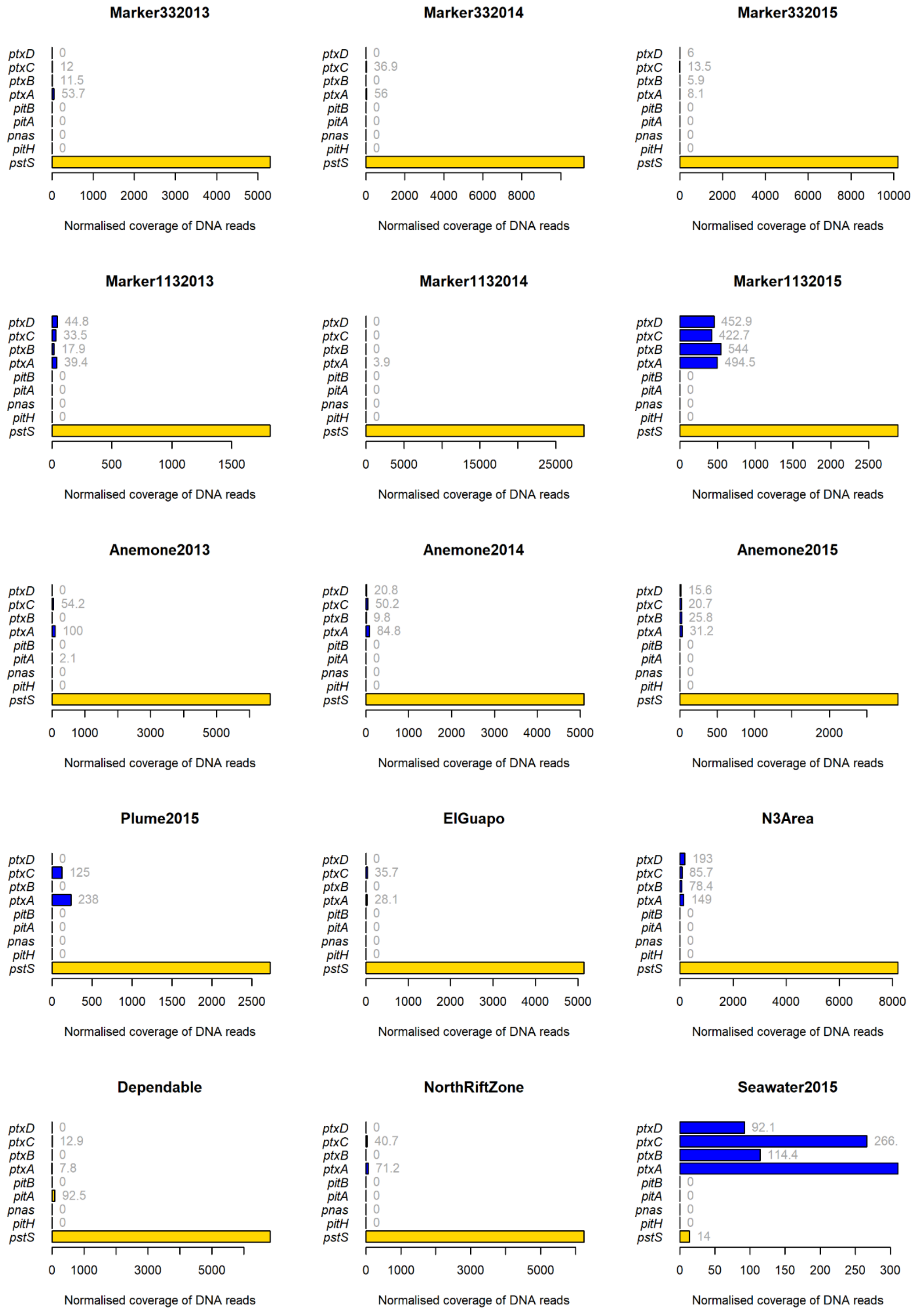

Figure S5: Relative coverage of genes for microbial phosphite utilization (blue; including import by *ptxA, ptxB* and *ptxC* as well as oxidation by *ptxD*) and phosphate uptake (yellow) at Von Damm.

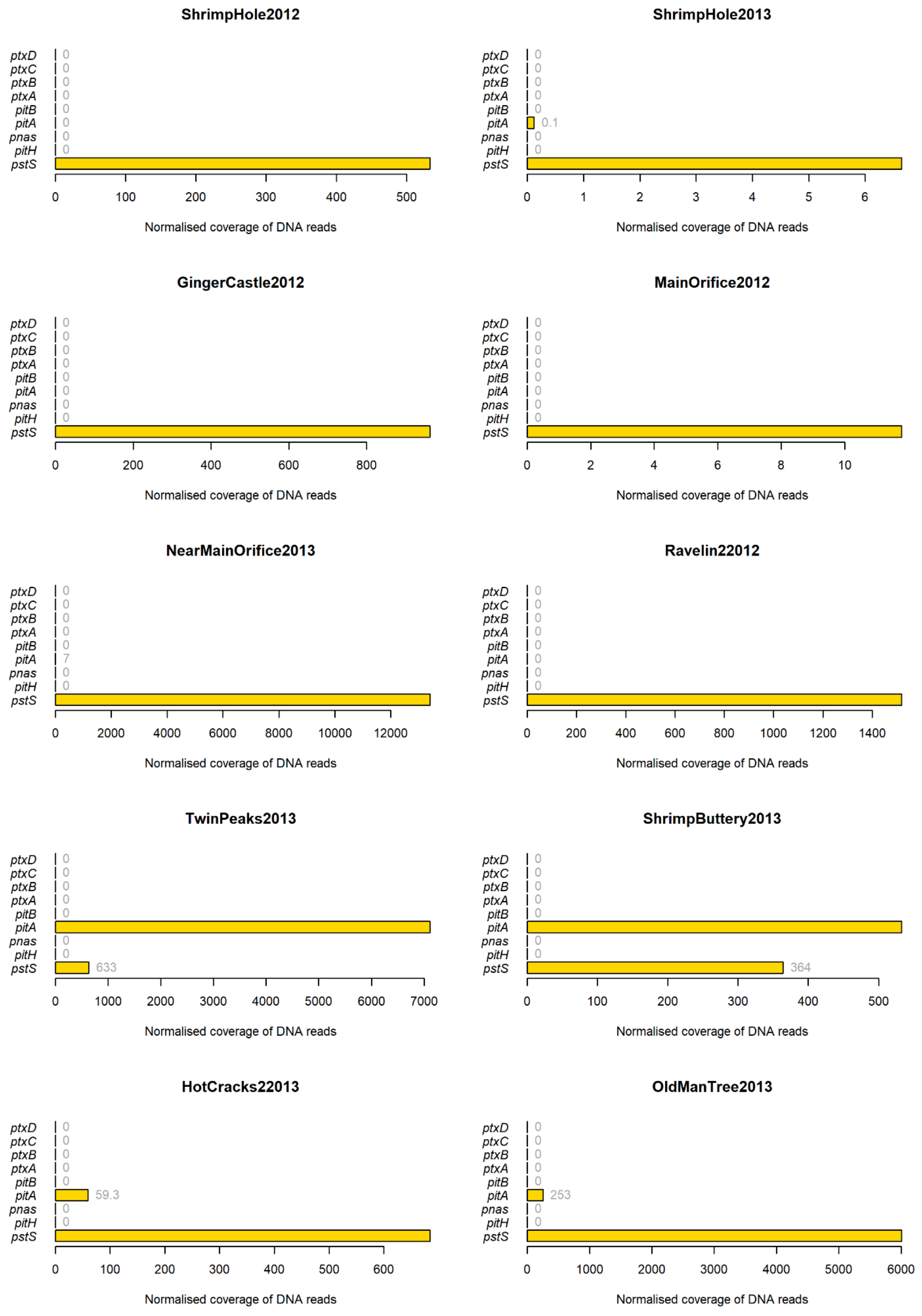

Figure S6: Relative coverage of genes for microbial phosphite utilization (blue; including import by *ptxA, ptxB* and *ptxC* as well as oxidation by *ptxD*) and phosphate uptake (yellow) at Lost City.

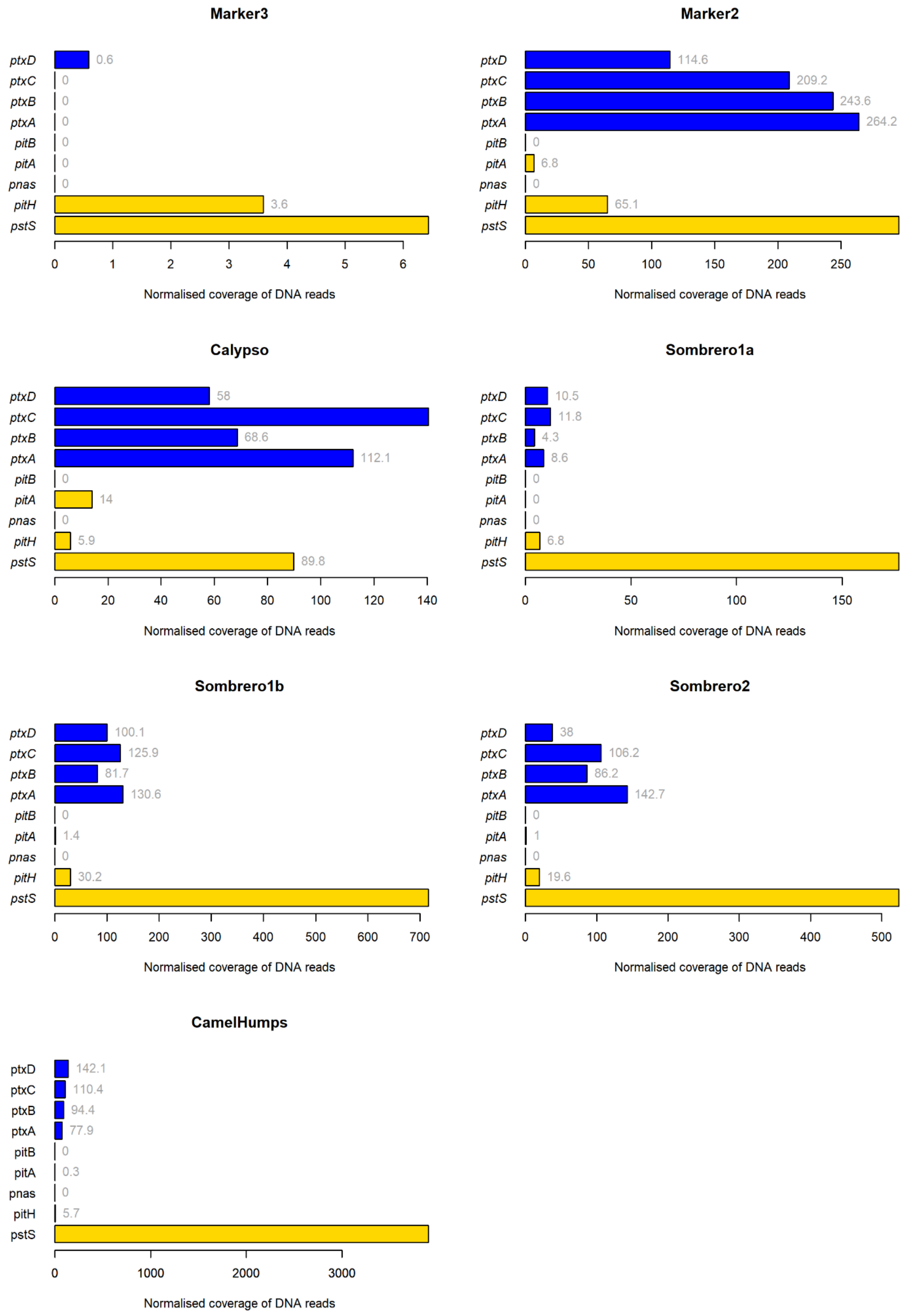

Figure S7: Relative coverage of genes for microbial phosphite utilization (blue; including import by *ptxA, ptxB* and *ptxC* as well as oxidation by *ptxD*) and phosphate uptake (yellow) at CROMO.

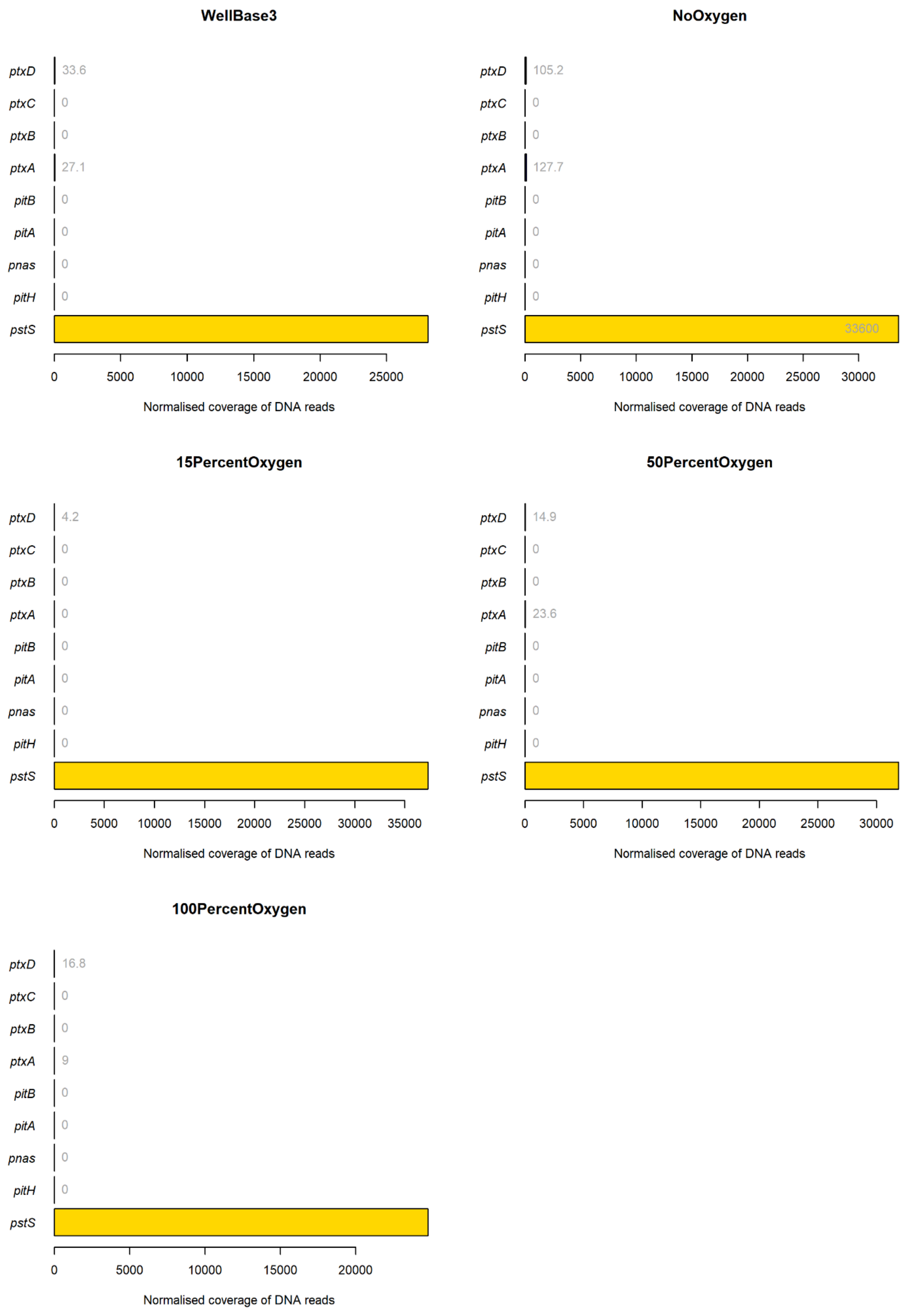

Figure S8: Relative coverage of genes for microbial phosphite utilization (blue; including import by *ptxA, ptxB* and *ptxC* as well as oxidation by *ptxD*) and phosphate uptake (yellow) in water samples from The Cedars.

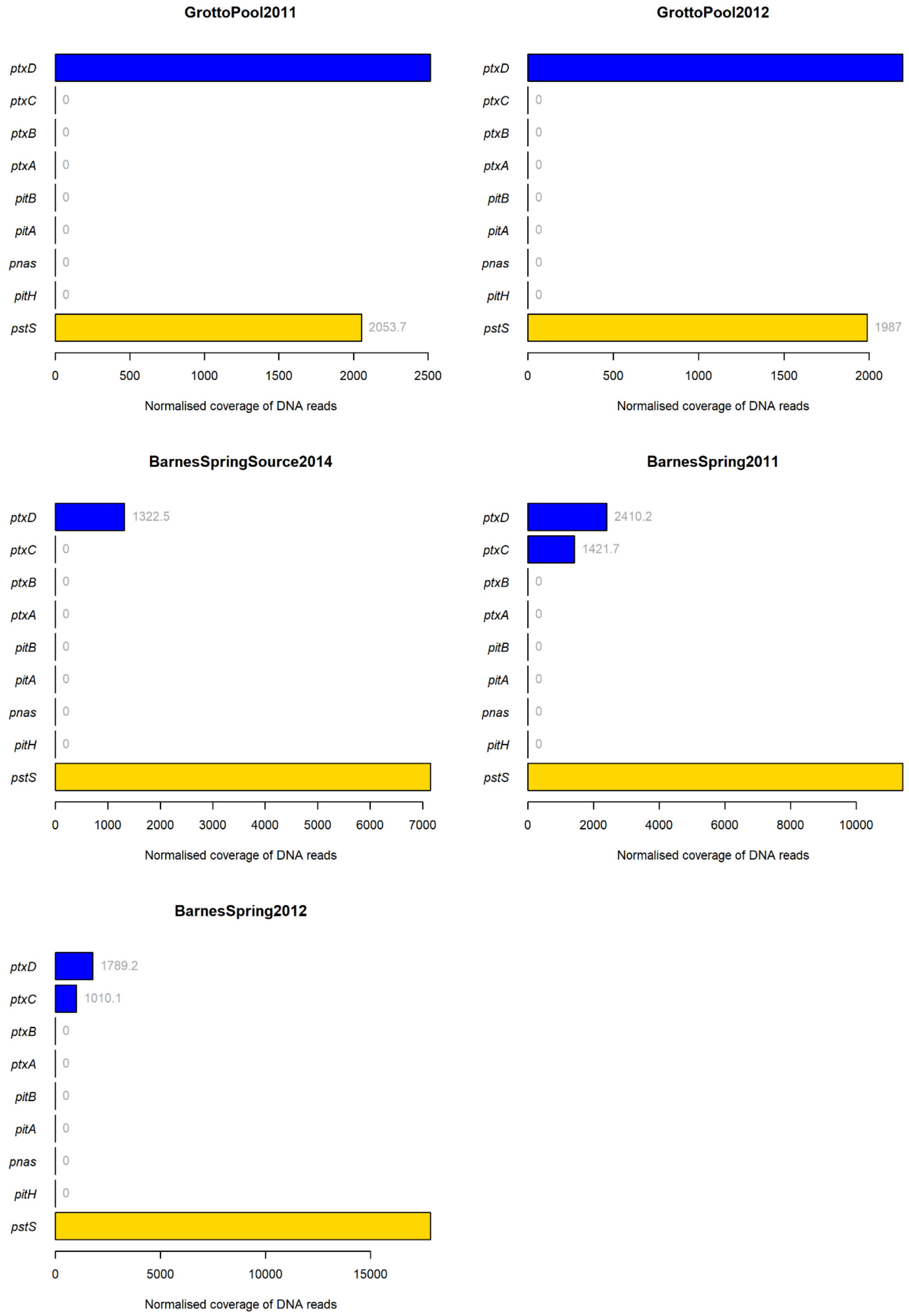

Figure S9: Relative coverage of genes and transcripts for microbial phosphite utilization (blue; including import by *ptxA, ptxB* and *ptxC* as well as oxidation by *ptxD*) and phosphate uptake (yellow) at Marker 2 and Sombrero1 vents in Lost City.

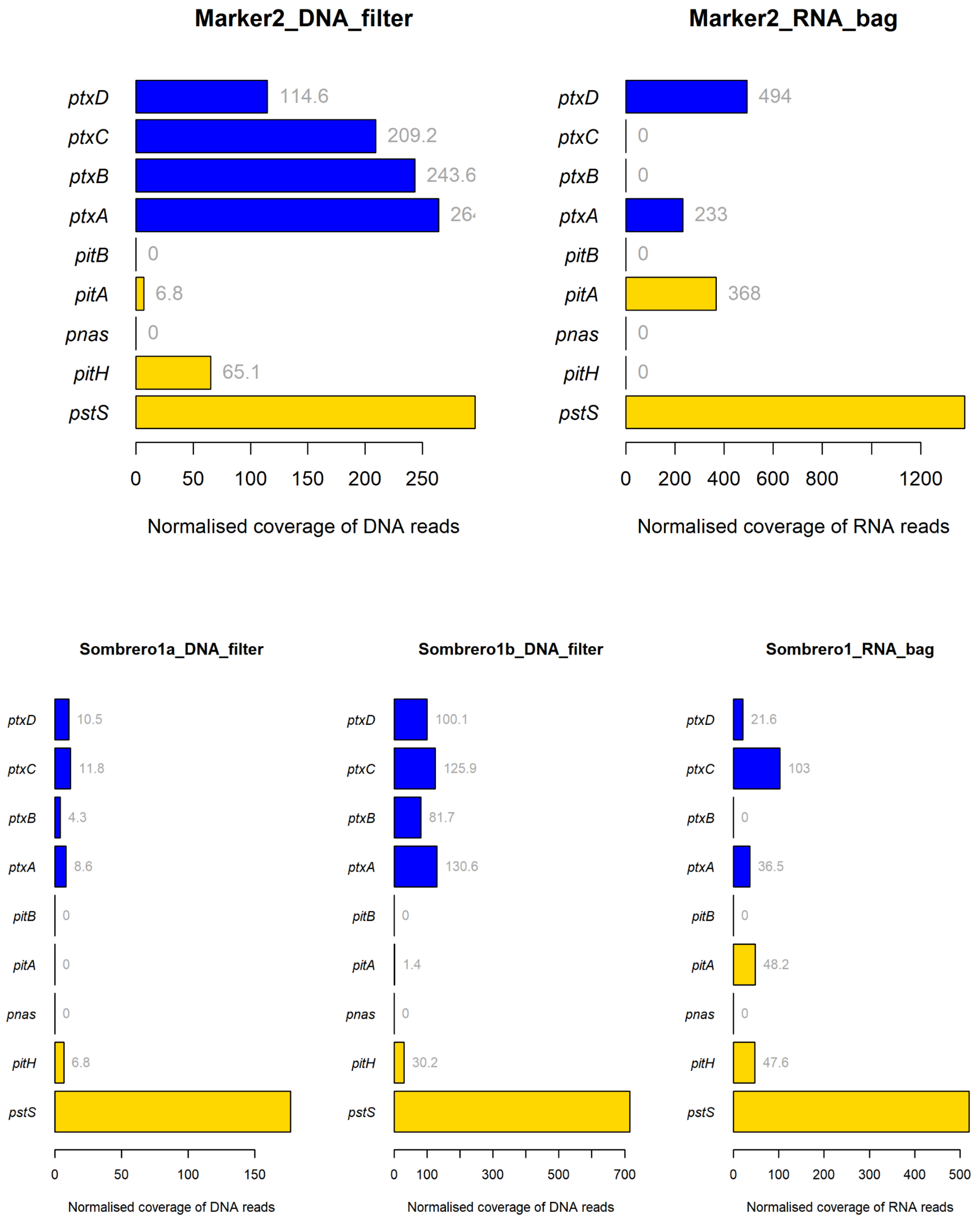

Figure S10: Relative coverage of genes for microbial phosphite utilization (blue; including import by *ptxA, ptxB* and *ptxC* as well as oxidation by *ptxD*) and phosphate uptake (yellow) at Piccard.

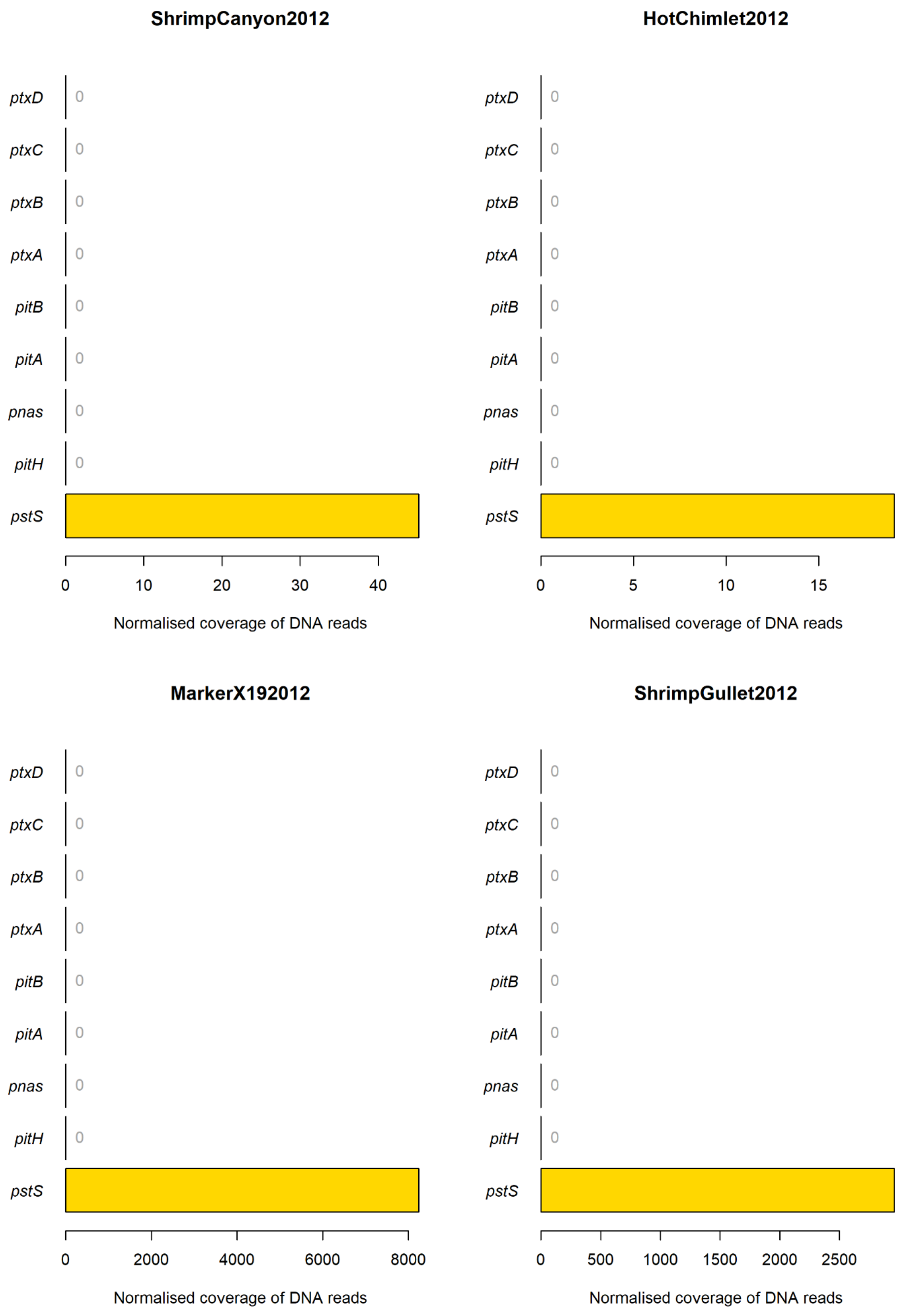

Figure S11: Relative coverage of genes for microbial phosphite utilization (blue; including import by *ptxA, ptxB* and *ptxC* as well as oxidation by *ptxD*) and phosphate uptake (yellow) at Voltri Massif.

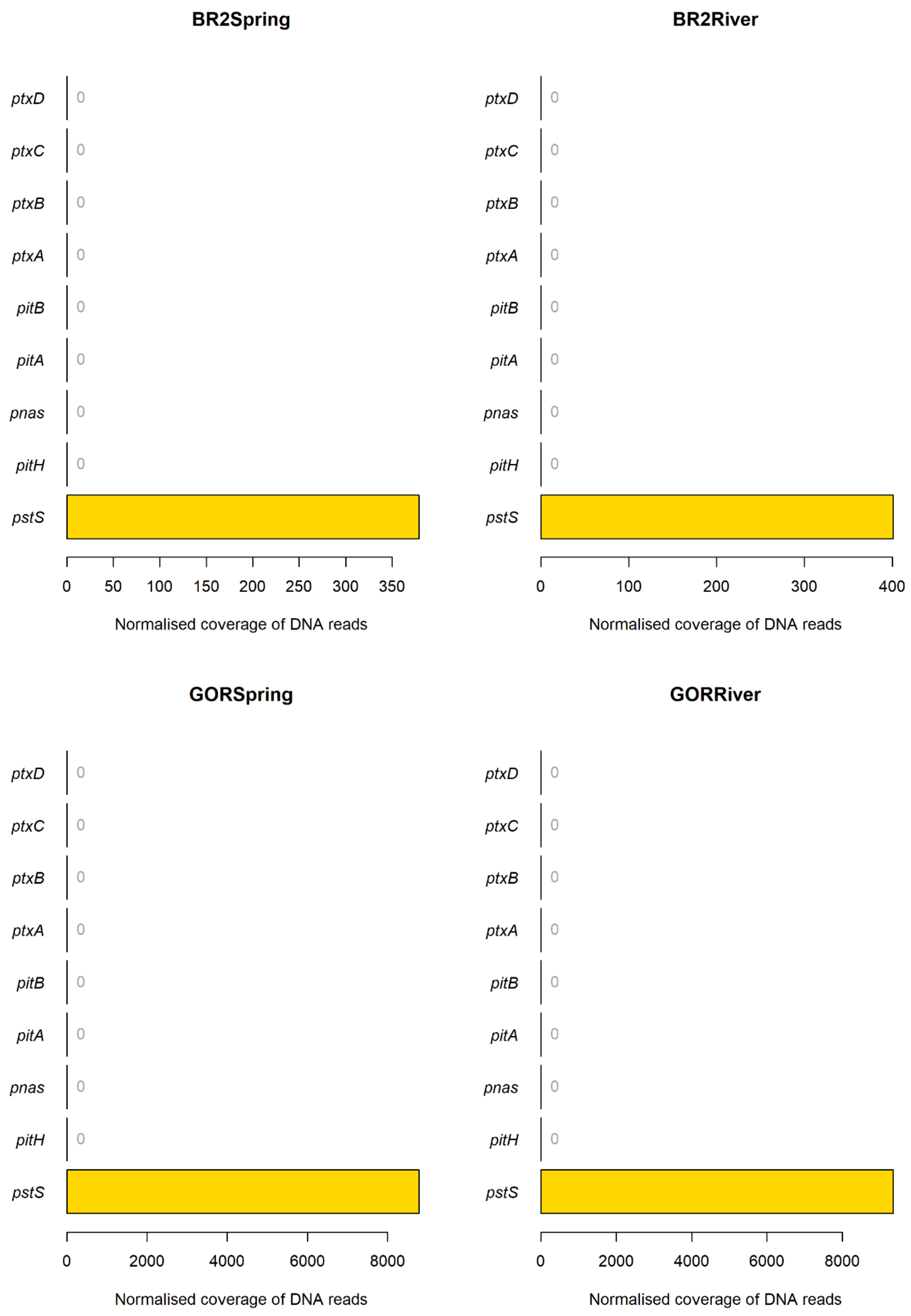

Figure S12: Relative coverage of genes for microbial phosphite utilization (blue; including import by *ptxA, ptxB* and *ptxC* as well as oxidation by *ptxD*) and phosphate uptake (yellow) in chimney samples from Prony Bay hydrothermal field.

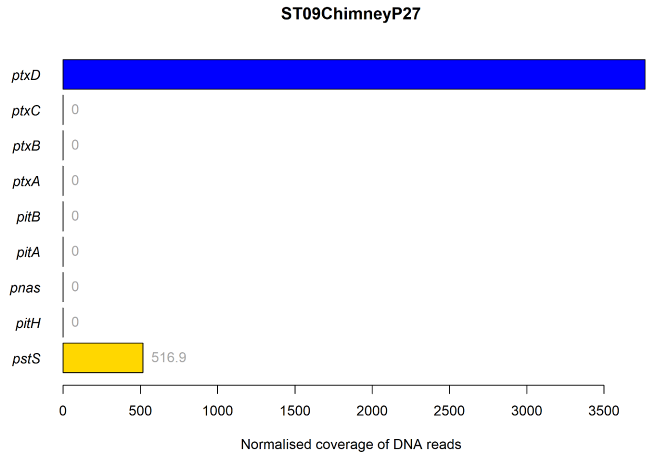

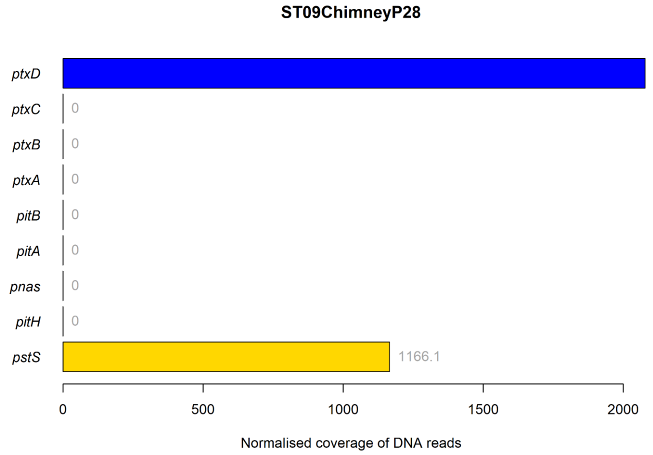

Figure S13: Relative coverage of genes for microbial phosphite utilization (blue; including import by *ptxA, ptxB* and *ptxC* as well as oxidation by *ptxD*) and phosphate uptake (yellow) in chimney samples from Old City.

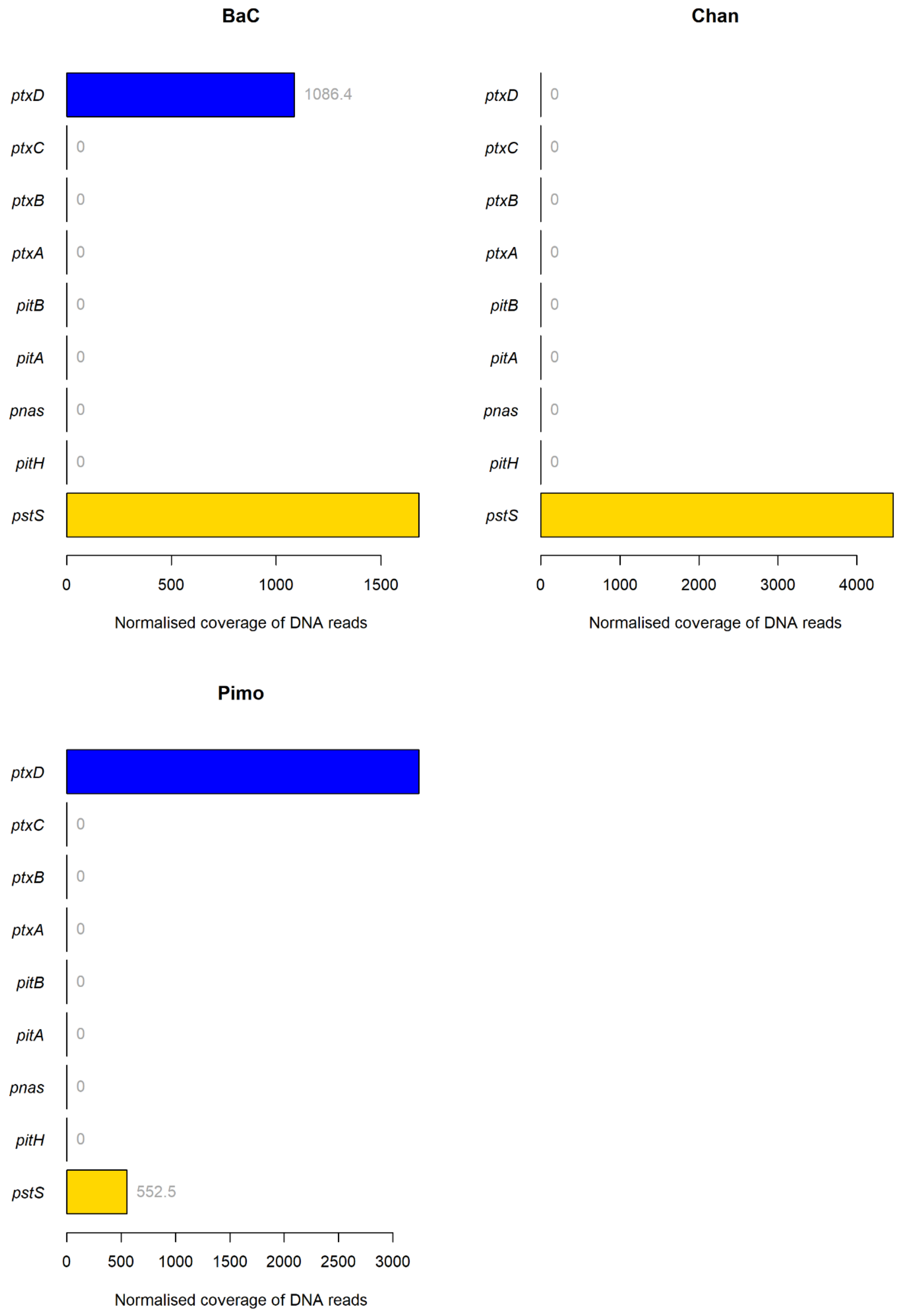

Supplement: Supplementary file 1 — Appendix S1. Tables and figures. [file GBI-23-e70016-s003.pdf]
